# Road rules for traffic on DNA—systematic analysis of transcriptional roadblocking *in vivo*

**DOI:** 10.1093/nar/gku627

**Published:** 2014-07-17

**Authors:** Nan Hao, Sandeep Krishna, Alexandra Ahlgren-Berg, Erin E. Cutts, Keith E. Shearwin, Ian B. Dodd

**Affiliations:** 1 School of Molecular and Biomedical Sciences (Biochemistry), The University of Adelaide, Adelaide, SA 5005, Australia; 2 Simons Centre for the Study of Living Machines, National Centre for Biological Sciences, Bangalore 560065, India

## Abstract

Genomic DNA is bound by many proteins that could potentially impede elongation of RNA polymerase (RNAP), but the factors determining the magnitude of transcriptional roadblocking *in vivo* are poorly understood. Through systematic experiments and modeling, we analyse how roadblocking by the *lac* repressor (LacI) in *Escherichia coli* cells is controlled by promoter firing rate, the concentration and affinity of the roadblocker protein, the transcription-coupled repair protein Mfd, and promoter–roadblock spacing. Increased readthrough of the roadblock at higher RNAP fluxes requires active dislodgement of LacI by multiple RNAPs. However, this RNAP cooperation effect occurs only for strong promoters because roadblock-paused RNAP is quickly terminated by Mfd. The results are most consistent with a single RNAP also sometimes dislodging LacI, though we cannot exclude the possibility that a single RNAP reads through by waiting for spontaneous LacI dissociation. Reducing the occupancy of the roadblock site by increasing the LacI off-rate (weakening the operator) increased dislodgement strongly, giving a stronger effect on readthrough than decreasing the LacI on-rate (decreasing LacI concentration). Thus, protein binding kinetics can be tuned to maintain site occupation while reducing detrimental roadblocking.

## INTRODUCTION

RNA polymerase (RNAP) inside cells transcribes DNA that is occupied by a variety of proteins, both static and mobile. Collisions between RNAP and other RNA and DNA polymerases have been intensively studied (1–3). However, most RNAP encounters must be with static DNA-bound proteins. Some DNA-bound proteins are known to form a roadblock to the progress of transcribing RNAP, strongly reducing transcription downstream of their binding site. However, despite the potentially large impact of transcriptional roadblocking, the factors that determine its strength *in vivo* are poorly understood.

The *Escherichia coli* *lac* repressor (LacI) can cause more than 80% reduction in transcription downstream of its binding site both *in vitro* and *in vivo* (4–6), and substantial reductions have also been observed for *E. coli* nucleoid-associated protein Fis (7), PurR and GalR repressors (8,9), as well as the *Bacillus subtilis* transcriptional regulators CcpA and CodY (10–12). In addition, LacI also impedes the progress of the major eukaryotic RNAP, PolII, *in vitro* and possibly *in vivo* (13,14). A DNA-cleavage-defective CRISPR protein–RNA complex can block transcription in *E. coli* and human cells (15,16). However, not all DNA-bound proteins can block the progression of RNAP. In studies of other transcription factors such as CI and CII of coliphage 186, *in vivo* roadblocking is almost absent (17,18). In addition, RNAP in eukaryotes or bacteria must frequently pass through nucleosomes or DNA bound by nucleoid-associated proteins.

*In vitro* studies have revealed some details of the processes occurring upon RNAP–roadblock encounters. Nucleosomes and various sequence-specific DNA binding proteins such as LacI, *Eco*RQ111 (a cleavage-defective form of EcoRI) and LexA cause a substantial pause in transcription of bacterial and eukaryotic RNAPs *in vitro* (4,19–22). Pausing can be associated with RNAP backtracking, in which RNAP and the associated DNA bubble move backward along the RNA and DNA chains, disengaging the 3′ end of the transcript from the catalytic site, forming long-lived inactive complexes *in vitro* and *in vivo* (23–25).

A fraction of RNAPs move past the roadblock site *in vitro*, though little readthrough is generally seen for *E. coli* RNAP and strong-binding roadblocks (5). It is not known whether readthrough is due to ‘escape’, in which RNAP takes advantage of spontaneous dissociation of the roadblock protein, or whether RNAP actively dislodges the roadblock. In the case of nucleosomes, it appears that a single RNAP can steadily unwind the DNA from the nucleosome, not by active displacement of DNA but by acting as a ratchet that prevents DNA rebinding after spontaneous unwrapping (26).

The extent of roadblocking *in vitro* and *in vivo* can be affected by accessory factors. Backtracked RNAP is rescued by GreA and GreB in *E. coli* and TFIIS in mammals, which stimulate cleavage of the RNA, regenerating a new 3′ end at the catalytic site (27), and can aid passage of RNAP through a LacI roadblock in *E. coli* cells (28). The transcription coupled repair (TCR) protein Mfd in *E. coli*, and Cockayne's Syndrome protein CSB in mammals, binds to the DNA behind RNAP and uses ATP to push backtracked RNAP forward until the 3′ end of RNA is back at the catalytic centre. However, the forces generated by Mfd may also result in RNAP termination (29,30). A terminator role for Mfd at protein roadblocks *in vivo* is supported by a decrease in roadblocking by CcpA, CodY and LacI in an *mfd* mutant (10,12,31). The *E. coli* RNA-binding termination factor Rho can also terminate RNAP stalled at roadblocks *in vitro* (21,32).

The presence of multiple paused RNAPs can increase passage through a protein roadblock *in vitro* and *in vivo*. This RNAP cooperation is proposed to be the major mechanism for overcoming roadblocks *in vivo* (5). A trailing RNAP can prevent backtracking and aid restart of a paused RNAP in front of it, but whether suppression of backtracking is sufficient to explain cooperation, or whether multiple RNAPs may also provide a combined ‘push’ to dislodge the roadblock, is not clear. A trailing ribosome can also help an RNAP overcome a LacI roadblock in bacteria (33). However, observations of pausing of trailing RNAPs at a protein roadblock site *in vivo* show that cooperation is not instantaneous (5,23). Under some conditions, multiple RNAPs can form a queue that extends back along the DNA to occlude or ‘clog’ the promoter (34).

It is unclear how these processes act in combination to determine roadblocking outcomes *in vivo*. It is not known under which conditions a roadblock will be strong or weak, and therefore how cells avoid excessive roadblocking in their genomes or how roadblocking can best be exploited for gene regulation. To address these questions, we have taken a systematic approach, combining quantitative experiments and mathematical modeling to dissect the impact of five factors on roadblocking by the LacI in live *E. coli* cells: (i) RNAP flux (promoter strength); (ii) roadblocker concentration; (iii) roadblocker affinity; (iv) Mfd; and (v) promoter–roadblock spacing. We used a modular, chromosomally integrated promoter–spacer–roadblock–*lacZ* reporter system to measure roadblocking and analysed the results by stochastic simulations. Our large body of observations was consistent with a relatively simple model of roadblocking and defined the critical role of the kinetics of the roadblocker protein.

## MATERIALS AND METHODS

### Strains, reporters and LacI expression constructs

All *lacZ* reporter constructs (Supplementary Figure S1 and Table S1) were integrated into the λ *attB* site of *E. coli* MG1655 *rph+ ΔlacIZYA*, or a derivative with an in-frame deletion of the *mfd* gene. Details of DNA constructions are given in Supplementary Data. Growth rate was similar in all strains.

### LacZ assays

Cells were grown at 37°C in minimal medium for microtiter plate-based LacZ assays as previously described (35). Background LacZ activities were measured via paired promoterless reporters (Supplementary Data).

### Stochastic modeling

**Figure 1.**
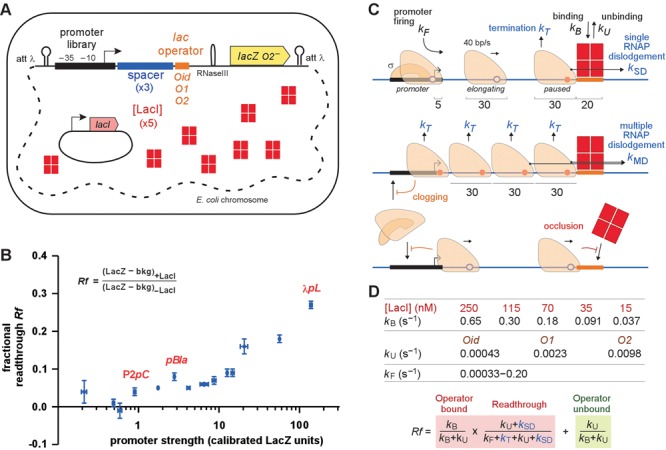
Measuring transcriptional roadblocking *in vivo*. (**A**) Modular, chromosomally integrated promoter–operator–*lacZ* chassis (see also Supplementary Figure S1) and constitutive LacI expression system for systematic variation of RNAP flux (promoter strength; Supplementary Table S1), promoter–roadblock spacing, LacI binding rate (LacI tetramer concentration) and LacI unbinding rate (*lac* operator affinity). (**B**) Readthrough fraction (*Rf*) for 15 promoters with the *Oid* operator, 250 nM LacI (tetramers) and a 102 bp promoter–operator spacer. Error bars show 95% confidence intervals (*n* = 9). (**C**) Processes and parameters included in the stochastic simulations. (**D**) Fixed parameter values (Supplementary Figure S2) and analytical equation for *Rf* in the absence of multiple RNAPs (see also Supplementary Figure S3).

Simulations of the processes of Figure 1C used a hybrid Gillespie/fixed time step algorithm derived from that of Sneppen *et al.* (36). A simulated annealing procedure was used to find values for *k_T_*, *k*_SD_ and *k*_MD_ that minimized the difference between simulated and measured *Rf*. Full details of the modeling are given in Supplementary Data. Parameter values are given in Supplementary Figure S2.

## RESULTS

### System for analysis of roadblocking *in vivo*

In our roadblocking assay system (Figure 1A), we used a series of 15 constitutive promoters upstream of the *lacZ* gene, including P2*pC*, *pBla*, λ*pL* and 12 synthetic promoters spanning a 611-fold range of activity (Supplementary Figure S1). Three different *lac* operators (*Oid*, *O1* and *O2*) were placed in the untranslated region between the promoter and *lacZ* (Figure 1A), avoiding effects of trailing ribosomes and varying the binding affinity of the roadblocking protein over a 23-fold range. Five concentrations of LacI over a 17-fold range were supplied from a medium-copy plasmid carrying the *lacI* gene and its wild-type promoter, or four promoter variants made by mutagenesis. LacI concentrations (Figure 1D) were measured by repression of a *plac.O2.lacZ* reporter (35) (Supplementary Data).

We measured the fractional readthrough, *Rf*, of the roadblock as the steady state LacZ units in the presence of LacI divided by the activity in the absence of LacI (backgrounds subtracted; Figure 1B). Thus *Rf* = 1 is complete readthrough or an absence of roadblocking, while *Rf* = 0 indicates no readthrough and 100% roadblocking efficiency.

Figure 1B shows the dependence of *Rf* on promoter strength with the ‘ideal’ *Oid* operator, the highest [LacI] (250 nM) and a 102 bp promoter–operator spacing. Roadblocking ranged from 96% for the weaker promoters (*Rf* = 0.04) to 73% (*Rf* = 0.27) for the strongest promoter, confirming the RNAP cooperation effect seen previously *in vitro* and *in vivo* (5,20). However, the relationship between *Rf* and RNAP flux was not simple, with substantial cooperation only appearing at high promoter strengths.

### Stochastic model of transcriptional roadblock

To extract quantitative information about the kinetic processes underlying roadblocking, we analysed our data using stochastic simulations that incorporate promoter firing, elongation, pausing and termination of RNAP, as well as binding and unbinding of the roadblocker (Figure 1C). Promoter firing was treated as a single step process that introduces an elongating RNAP at an empty promoter, occurring with a fixed rate *k_F_* (s^−1^). This and any other elongating RNAP on the promoter–spacer–operator DNA segment were advanced by 1 bp in each time step (1/40 s) (36). Although stochastic RNAP translocation, pausing and backtracking can affect RNAP progress and interaction (37,38), we think these effects will be small over the short distances we used. We used 30 bp as the space occupied by an elongating RNAP, the region occupied in the crystal structure of elongating *Thermus thermophilus* RNAP (39), and consistent with nuclease measurements (21,32). RNAP binding at the promoter is assumed to be possible if no other RNAP overlaps the +5 position.

In the model, an RNAP became paused if it met the bound roadblocker or another paused RNAP. A paused RNAP either remained paused, was terminated (removed) with a rate *k_T_* or moved forward to dislodge the roadblock protein (Figure 1C). We allowed a single RNAP to dislodge the roadblock with rate *k*_SD_ (single dislodgement), while if multiple RNAPs were queued at the roadblock, a different rate *k*_MD_ (multiple dislodgement) was applied to allow for RNAP cooperation. Note that we did not explicitly model backtracking at the roadblock. Binding of the LacI roadblocker to its 20 bp operator occurred with a rate *k_B_* (s^−1^) as long as the operator was free of RNAP. The same *k_B_* value was used for each of the three *lac* operators, and was calculated as the product of the LacI concentration and its on-rate constant 2.51 × 10^6^ M^−1^s^−1^, obtained from *in vivo* imaging experiments estimating a single LacI tetramer in the cell takes ∼4 min to find its operator (40–42). The five LacI concentrations allowed us to vary the LacI binding rate *k_B_* over a 17-fold range (Figure 1D). The dissociation of LacI from its operator occurred with a rate *k_U_* (unbinding, s^−1^) (Figure 1D), calculated from its on-rate constant and *in vivo* dissociation constants measured for *Oid*, *O1* and *O2* (43).

We determined ‘relative’ promoter firing rates for our promoters using reporter expression in the absence of LacI (Supplementary Figure S1). To obtain estimates of ‘absolute’ firing rates (Supplementary Table S1), we included in our promoter series the λ*pL* and *pBla* promoters, for which *in vivo* firing rates have been estimated under different growth conditions by comparison with ribosomal RNA promoters (44).

**Figure 2.**
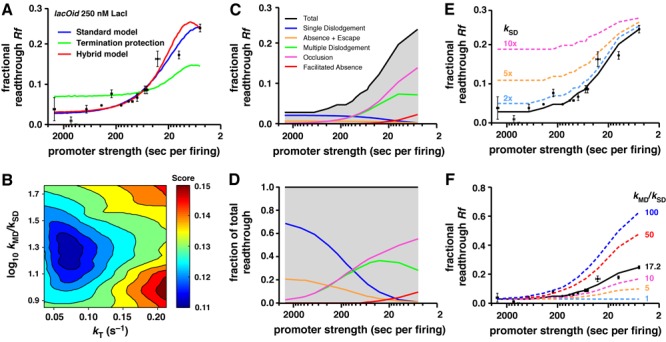
Stochastic modeling of transcriptional roadblocking. (**A**) *Rf* values versus firing rate for the 15 promoters (Supplementary Figure S1 and Supplementary Table S1). The blue curve shows the average *Rf* from simulations of the standard model, where all paused RNAPs are subject to termination (*k_T_* = 0.066, *k*_SD_ = 0.0015 and *k*_MD_ = 0.026 s^−1^). Simulation of the pure termination protection model (termination is blocked by a trailing RNAP and *k*_SD_ = *k*_MD_; green curve) and a hybrid termination protection model (termination is blocked by a trailing RNAP and *k*_MD_ > *k*_SD_; red curve). (**B**) A portion of the fitting optimization landscape (Supplementary Data) for *k_T_* and the ratio of the dislodgement rates (*k*_MD_/*k*_SD_) for the standard model. The optimal *k*_MD_/*k*_SD_ ratio was 17.2. All three fitted parameters converged on clear optimal values. See also Supplementary Figure S4. (**C**,**D**) Absolute (C) and fractional (D) contributions to readthrough of dislodgement (single and multiple), absence + escape, occlusion and facilitated absence (see text for definitions). (**E**) Simulations with 2- to 10-fold increases in *k*_SD_ over 0.0015 s^−1^ (fixed *k*_MD_ = 0.026 s^−1^) predict larger increases in *Rf* for weak promoters than for strong promoters. (**F**) Simulations with altered *k*_MD_/*k*_SD_ ratios (fixed *k*_SD_ = 0.0015 s^−1^) show that cooperation requires *k*_MD_ > *k*_SD_.

Readthrough (*Rf*) was determined in the simulations by comparing the number of RNAPs passing the operator per unit time in the presence of LacI with the number passing in its absence. Simulations were repeated in a Monte Carlo simulated annealing approach to find values of *k_T_*, *k*_SD_ and *k*_MD_ that could best reproduce the observed *Rf* versus promoter strength data for the *Oid* roadblock with 250 nM LacI. The model gave a reasonable fit to the data (Figure 2A, blue curve), with fitting converging on clear optimal values: *k_T_* = 0.066 s^−1^, *k*_SD_ = 0.0015 s^−1^ and *k*_MD_ = 0.026 s^−1^ (Figure 2B and Supplementary Figure S4).

These fitted parameter values are not strongly sensitive to variation in the values used for size or speed of RNAP. However, they are affected by variation in the calibration of absolute promoter firing rates and by variation in the parameters specifying the kinetics of LacI unbinding (Supplementary Data; Supplementary Figures S5 and S6; also discussed below).

### Does a single RNAP actively dislodge a LacI roadblock *in vivo*?

The non-zero value obtained for *k*_SD_ in the stochastic model means that the best fit is obtained with a single RNAP being able to actively dislodge the LacI roadblock at *Oid*, that is, increasing its rate of unbinding. While some effect of a single RNAP on roadblock dissociation seems intuitive, direct evidence for dislodgement by a single RNAP has been lacking.

The need for dislodgement by single RNAP to explain our results can be understood using an analytical model that can be applied when the promoter is exceedingly weak (Figure 1D and Supplementary Figure S3). In this case, there will almost never be two RNAPs queued at the roadblock, since the rates of termination and dislodgement far exceed the firing rate, and the first RNAP will either get through the roadblock or terminate before a second RNAP arrives. Our weakest promoter fires every 3016 s (*k_F_* = 0.00033 s^−1^), 220-fold slower than *k_T_*. Thus, the ∼4% *Rf* seen with this promoter is due to three processes: (i) ‘dislodgement’ of the roadblock by a single RNAP, (ii) ‘escape’ due to spontaneous roadblocker unbinding that allows an RNAP paused at the roadblock to resume elongating, and (iii) ‘avoidance’ of the roadblock, where the RNAP arrives at an unoccupied operator. Avoidance must be exceedingly rare in our experiment, since *Oid* is only *k_U_*/(*k_B_*+*k_U_*) = 0.07% unoccupied (Figure 1D). Escape due to LacI unbinding is only capable of giving at most ∼*k_U_*/(*k_T_*+*k_U_*) = 0.67% readthrough (Figure 1D; setting *k*_SD_ = 0). Thus, the majority of the readthrough seen must be due to single RNAP dislodgement. This was confirmed by our simulations, where for weak promoters, dislodgement of the roadblocker by a single RNAP contributed to 73% of the transcripts passing the *lac* operator (Figure 2C and D).

However, this conclusion is dependent on the fixed values used for the rate of promoter firing and the rate of spontaneous LacI unbinding (Supplementary Figure S6; Supplementary Data). It is possible for the model to give a reasonable fit to the data with *k*_SD_ = 0, if the true *k_F_* values are more than 5-fold lower than our calibration with literature measurements (44) indicates. A *k*_SD_ = 0 can also be obtained if *k_U_* is ∼6-fold higher than estimated, that is, if LacI binding–unbinding kinetics are substantially faster than obtained from *in vivo* imaging experiments (40). Lower RNAP fluxes and more rapid LacI kinetics mean that readthrough due to escape increases such that dislodgement by a single RNAP is not necessary to explain readthrough at low promoter firing rates. The uncertainty in these literature values is difficult to ascertain, thus, while existing measurements support dislodgement of LacI by a single RNAP, they are not conclusive in this regard.

### Multiple RNAPs increase roadblock dislodgement

A number of mechanisms might cause the increase in readthrough with increased RNAP flux (Figure 2A). Multiple RNAPs may increase active dislodgement of the roadblock, the high density of elongating RNAPs might block access of LacI to its operator, or a trailing RNAP queued behind the leading RNAP paused at the roadblock might protect it from termination.

The modeling shows that increased dislodgement by multiple RNAPs is needed to reproduce the cooperation seen, with the rate of LacI dislodgement by multiple RNAPs (*k*_MD_) estimated to be ∼17-fold higher than by a single RNAP (*k*_SD_; Figure 2B). If *k*_MD_ = *k*_SD_, then no cooperation is seen (Figure 2F). The contribution of single dislodgement to the readthrough becomes negligible as the firing rate increases (Figure 2C and D), such that increasing the *k*_SD_ increases the readthrough by weak promoters but not by strong promoters (Figure 2E).

The RNAP-density mechanisms for cooperation alter the magnitude of readthrough but their effect is largely dependent on multiple RNAP dislodgement. We distinguish two mechanisms, ‘occlusion’ and ‘facilitated absence’. Occlusion occurs when the next RNAP behind an RNAP that has just traversed the operator follows so closely that there is no room for LacI to bind to the operator. The occlusion effect is large (Figure 2C and D) primarily because after a multiple dislodgement event it allows all the trailing RNAPs that were previously paused in the queue to pass the operator. Even without this ‘free pass’ occlusion by the queue, the gaps between successive ‘unpaused’ RNAPs at high flux can in theory become small enough to make it impossible for LacI to bind. However, for the required gap of at least 1.25 s between successive RNAP fronts (20 bp operator; 30 bp RNAP; RNAP velocity 40 bp/s), the equation *p*(operator free) = 1/exp(gap/(1/*k_F_*)) (36) indicates that even for λ*pL* (*k_F_* = 0.2 s^−1^), the *lac* operator is unoccluded 78% of the time. Thus, this non-queue-associated occlusion effect is small.

Facilitated absence occurs when the gap between an RNAP crossing the operator and the next RNAP is large enough that LacI ‘could’ bind but does not do so before the second RNAP reaches the operator. For *Oid* and 250 nM LacI, this effect is small (Figure 2C and D) because at this concentration LacI binding takes on average 1/*k_B_* ∼ 1.5 s, and even for the fastest firing promoter, λ*pL*, the gaps between successive RNAPs are on average 4.2 s (5–30/40 s).

An alternative mechanism for cooperation that is not part of our standard model is that trailing RNAPs might promote readthrough by protecting the leading RNAP from termination. For example, Mfd requires access to ∼25 bp of DNA upstream of the RNAP for its action *in vitro* (30), and this access could be blocked by a trailing RNAP. We examined whether a termination protection mechanism alone could explain the cooperation effect seen in the *Oid* 250 nM LacI data by allowing only the promoter proximal paused RNAP to be subject to termination, and fixing *k*_MD_ = *k*_SD_. We were unable to obtain a good fit to the data with a pure termination protection model due to insufficient cooperation (Figure 2A, green curve). However, combining termination protection with the *k*_MD_ > *k*_SD_ mechanism allowed a good fit to the data with only a moderate increase in *k_T_*, and reduced the optimal *k*_MD_ to about 6.5-fold stronger than *k*_SD_ (Figure 2A and Supplementary Figure S2). Thus, termination protection may contribute to RNAP cooperation but it cannot substitute for increased dislodgement by multiple RNAPs.

### Reducing the roadblocker concentration increases RNAP cooperation by facilitated absence

**Figure 3.**
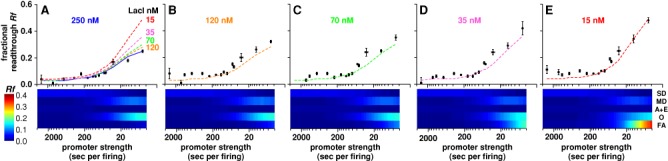
Effect of altering LacI binding rate on transcriptional roadblocking. (**A**) Data and simulation (solid curve) for *Oid* roadblock with 250 nM LacI. The dashed curves show predicted *Rf* for each of the four reduced LacI concentrations, obtained by simulations with reduced *k_B_* values. (**B**–**E**) Observed and predicted (dashed curves) *Rf* at four lower LacI concentrations. Error bars are 95% confidence intervals (*n* = 9). Heatmaps below each plot show the contribution of different readthrough mechanisms to the *Rf* in simulations: SD, single dislodgement; MD, multiple dislodgement; A+E, absence and escape; O, occlusion; FA, facilitated absence.

We used the model to predict the effect of changing the LacI concentration on readthrough. Changing LacI concentration changes *k_B_*, the rate at which an empty operator is filled by LacI. Decreasing *k_B_* substantially increased readthrough for fast firing promoters, but only marginally affected slow firing promoters (Figure 3A). *Rf* measurements at these different levels of LacI were in good agreement with the model, showing an increased RNAP cooperation effect (Figure 3A–E).

The lack of effect of reduced LacI concentration on slower promoters indicates that the increased readthrough for strong promoters is not simply due to low natural occupation of the operator by LacI (the absence mechanism). Even at 15 nM LacI, *Oid* is still 98.9% occupied (in the absence of RNAP). Instead, the simulations indicate that this increased readthrough is due mainly to facilitated absence, where RNAPs get through before LacI can rebind after a dislodgement event (Figure 3A–E). For λ*pL*, as much as 60% of the readthrough of *Oid* at 15 nM LacI is due to this mechanism (Figure 3E). At 15 nM LacI (*k_B_* = 0.037 s^−1^), the average time for LacI rebinding is ∼27 s. Thus, at high firing rates, a LacI dislodgement will lead to additional RNAPs passing the operator before it refills.

Repeating the parameter fitting using the data from all five LacI concentrations (Figure 3A–E and Supplementary Figure S7A) gave optimal values for *k_T_*, *k*_SD_ and *k*_MD_ similar to those obtained from fitting of the 250 nM LacI concentration alone (Supplementary Figure S2). Since these parameters are derived from a larger data set, they were used in subsequent modeling.

### Decreasing the roadblocker–DNA affinity increases readthrough by dislodgement

**Figure 4.**
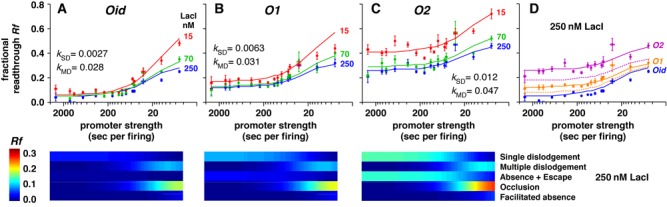
Effect of altering LacI unbinding rate on transcriptional roadblocking. (**A**,**B**,**C**) *Rf* data for *Oid* (A), *O1* (B) and *O2* (C) with 250, 70 and 15 nM of LacI. Error bars show 95% confidence intervals (*n* = 9). The solid curves are simulated *Rf* from global fitting with all five concentrations of LacI (data and fitting for 120 and 35 nM LacI are shown in Supplementary Figure S7). In the fitting, termination rate (*k_T_*) was the same for all operators, while different dislodgement rates (*k*_SD_ and *k*_MD_) were allowed (see also Supplementary Figure S2). Panels below each graph show the ‘absolute’ contribution of different readthrough mechanisms to the *Rf* at 250 nM LacI for the three operators. (**D**) If the dislodgement rates were kept the same as *Oid*, the model leads to an underestimation of *Rf* for *O1* and *O2* (dashed lines) in comparison to the optimal fitting (solid lines).

To test how the affinity of the roadblocker protein for its binding site would affect roadblocking, we replaced the high affinity *Oid* operator with lower affinity *O1* or *O2* operators, which have ∼5.2-fold and ∼22.9-fold increased *in vivo* dissociation constants, respectively (43). Assuming that the on-rate constant for LacI (*k*_on_) is the same for different operators, the effect of these DNA sequence changes is to increase the rate of LacI unbinding from the operator, *k_U_*. We found large increases in readthrough with decreasing LacI–operator affinity for both slow- and fast-firing promoters (Figure 4 and Supplementary Figure S7).

Increased *k_U_* might increase readthrough through three mechanisms: increased roadblock absence, increased escape due to spontaneous unbinding and increased dislodgement by RNAP. To determine the contribution of these mechanisms, we fitted the *O1* and *O2* data, allowing distinct values of *k*_SD_ and *k*_MD_ for each operator, while keeping *k_T_* fixed.

Unlike the case for the *Oid* operator, where readthrough by roadblock absence and escape was rare, these processes contribute substantially to readthrough for *O1* (27% of *Rf* = 12%) and *O2* (47% of *Rf* = 25%) for weak promoters at 250 nM LacI (Figure 4A–C). However, the model indicates that increased absence and escape alone are not enough to reproduce the increased readthrough seen for *O1* and *O2* (Figure 4D). The best fits gave progressive increases in *k*_SD_ and *k*_MD_ as the operator affinity decreased, leading to increasing single and multiple dislodgement and associated occlusion of the operator by the queued polymerases (Figure 4A–C and Supplementary Figure S7). As for *Oid*, we note that if promoter firing rates are slower than we estimate, or if LacI kinetics are faster than we estimate, then reasonable fits can be obtained with *k*_SD_ = 0.

### Mfd is the major factor responsible for removing roadblock-stalled RNAP

Optimal fits to our experimental data were obtained with the rate of termination *k_T_* of 0.063 s^−1^. Thus, an RNAP stalled at the roadblock or blocked by a stalled leading RNAP has about a 1-in-16 chance per second of being terminated *in vivo*, equivalent to a half-life of ∼11 s ( = ln2/*k_T_*).

**Figure 5.**
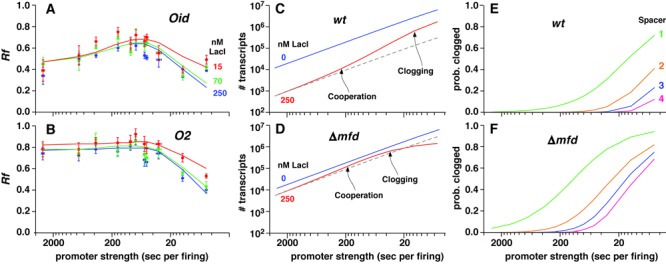
Effect of Δ*mfd* on transcriptional roadblocking. *Rf* values for 11 promoters and a *Oid* (**A**) or *O2* (**B**) roadblock in Δ*mfd* cells with 250, 70 and 15 nM of LacI. *Rf* values were obtained ±LacI as for *mfd*^+^ cells, with promoterless controls (also ±LacI) used to obtain background activities. Error bars are 95% confidence intervals (*n* = 9). The solid curves show simulations from global fitting of the data with a single termination rate (*k_T_*) and separate *k*_SD_ and *k*_MD_ for each operator. All fitted parameters converged on clear optimal values (Supplementary Figure S8C). (**C**,**D**) Modeled effect of cooperation and promoter clogging on transcription in *wt* (C) and Δ*mfd* (D) cells with *Oid* and 250 nM LacI, showing the limits of cooperation in eliminating roadblocking. (**E**,**F**) The predicted proportion of time that different length promoter–roadblock spacer sequences (able to accommodate one, two, three or four RNAPs) become ‘saturated’ with stalled RNAP and clog the promoter in both *wt* (E) and Δ*mfd* (F) cells.

To quantitate the role of Mfd in this termination, we constructed a *Δmfd* reporter strain and repeated our assays using 11 promoters, two *lac* operators and three LacI concentrations. Deletion of *mfd* had a strong effect on roadblocking (Figure 5A and B). For the weakest promoters, readthrough increased substantially to ∼40% for *Oid* (compared to ∼5% for *mfd^+^*) and ∼80% for *O2* (compared to ∼25–40% for *mfd^+^*). Strikingly, the effect of increasing promoter strength was quite different to the *mfd^+^* case, with a decrease in *Rf* seen at the highest promoter activities.

These data could be well fitted by the stochastic model by reducing the rate of termination, combined with small decreases in *k*_SD_ and *k*_MD_ (Figure 5A and B, Supplementary Figures S2 and S8C). The best fit was with *k_T_* = 0.0045 s^−1^, a 14-fold reduction compared with *mfd^+^*, which implies that 90% of RNAP termination at the roadblock in our system is due to Mfd. The 2–4-fold decreases in fitted *k*_SD_ and *k*_MD_ values in *Δmfd* cells (Supplementary Figure S2) suggest that forward translocation of the stalled RNAP by Mfd may sometimes lead to dislodgement of the roadblock without termination.

The simulations indicate that the decreasing readthrough at high promoter strengths in the *Δmfd* cells is due to promoter clogging, where the queue of RNAPs builds up at the roadblock and extends backward along the DNA, eventually preventing RNAP binding to the promoter. Once the RNAP flux is sufficient to clog the promoter, the rate of RNAP passage past the roadblock cannot increase (Figure 5D). In our system, promoter clogging limits the effect of cooperation, such that increased promoter strength can never completely overcome a strong roadblock. Clogging occurs at our highest *k_F_* values even in *mfd^+^* cells but is exacerbated when termination is reduced (Figure 5C and D).

Clogging should become more significant as the roadblock is moved closer to the promoter. The predicted probability of clogging for the 102 bp spacer (able to accommodate four RNAPs) is quite low, even for the highest promoter strengths, but increases substantially with shorter spacers (Figure 5E). However, for weak promoters, clogging is infrequent, even with very short spacers, except in *Δmfd* cells (Figure 5E and F). In *Δmfd* cells, significant clogging of the 102 bp spacer occurs with both strong and intermediate promoters (Figure 5D, Supplementary Figures S8A and B).

### The effect of reduced distance between the promoter and the roadblock

**Figure 6.**
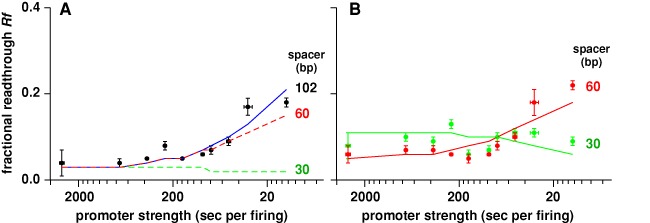
Effect of reducing promoter–operator spacing on transcriptional roadblocking. (**A**) *Rf* values of 10 promoters 102 bp upstream of a *Oid* roadblock, with 250 nM of LacI (model fit in blue). The dashed curves show simulated *Rf* from 60 bp and 30 bp promoter–operator spacer using the standard model parameters. Note that the strongest promoter λ*pL* is excluded from this analysis as λ*pL* extends from −73 to +90, precluding its use in short promoter–operator spacer experiments. (**B**) *Rf* for 60 bp and 30 bp promoter–operator spacers. Error bars are 95% confidence intervals (*n* = 9). The solid curves show the optimal fits from stochastic simulation, obtained from fitting the data with the same termination rate (*k_T_*) as for 102 bp promoter–operator spacer and increased dislodgement rates (Supplementary Figure S2).

Our modeling (Figures 5E and 6A) predicts that reducing the distance between the promoter and the roadblock from a 4-RNAP spacing to a 2-RNAP spacing should have little effect on the queue length in wild-type cells and should thus not substantially alter the amount of cooperation by multiple RNAPs or the amount of promoter clogging. In contrast, a 1-RNAP spacing should eliminate cooperation, preventing any increase in *Rf* with increasing promoter strength. A 1-RNAP spacing should also lead to promoter clogging and reduced *Rf* at high promoter strengths.

To test this, we shortened the spacer between the transcription start site and the edge of *Oid* to either 60 bp (2-RNAP) or 30 bp (1-RNAP) and measured readthrough using 10 promoters and 250 nM LacI (Figure 6B). As predicted, the effect of shortening to the 60 bp spacer was small, though readthrough was increased for the weaker promoters. However, the results for the 1-RNAP spacer were more complex (Figure 6B). The cooperation effect was indeed lost as predicted, that is, there was no increase in readthrough with increasing promoter strength. Unexpectedly, the readthrough values for the weaker promoters were systematically much higher than for the longer spacers. In addition, there was no observable clogging effect.

A possible explanation of the increased readthrough is that the dissociation of the RNAP σ^70^ subunit from the elongating complex takes some time (45,46), and the presence of σ^70^ is known to interfere with Mfd action *in vitro* (30). In the 30 bp and 60 bp constructs, the RNAP can travel at most 25 bp or 55 bp before meeting the roadblock. Thus, at least some of the stalled RNAPs may retain σ^70^ for some time and be resistant to Mfd termination. We found that reduced termination could allow the simulations to reproduce the 60 bp spacer data reasonably well. But the reductions in termination required to reproduce the high readthroughs for the weaker promoters at the 30 bp spacer produced strong clogging and reduced readthrough at high promoter strengths. Instead, reasonable fits to the data for both the 60 bp and 30 bp spacers could be obtained if dislodgement rates by single RNAP (*k*_SD_) were increased some 2.1-fold and 5.3-fold, respectively (Figure 6B and Supplementary Figure S2).

## DISCUSSION

### A simple model for transcriptional roadblocking

The effects on roadblocking of four of the five parameters we examined—RNAP flux (*k_F_*), roadblocker concentration (*k_B_*), roadblocker unbinding (*k_U_*) and termination (*k_T_*)—can be well explained by remarkably straightforward mechanisms of RNAP–roadblock interaction (Figure 1C). The effect of the fifth parameter—promoter–roadblock spacing—did not fully conform to the expectations of the model in the case of the shortest spacing, where readthrough was higher than expected. The modeling suggested that this result is not consistent with decreased termination of an RNAP stalled close to the promoter, but could be explained if a single promoter-proximal RNAP is more likely to dislodge the roadblock than a promoter-distal RNAP. We speculate that this may be due to sequence differences at the different stall sites (e.g. differential backtracking), which we have not controlled for. Alternatively, the RNAP may be different. The σ^70^ subunit is lost with a half-life of ∼7 s (46) and various elongation regulating factors (e.g. NusA, NusG, rho) bind to the RNAP after it leaves the promoter (47). Such changes could decrease the ability of the RNAP to dislodge the roadblock. However, the 72 bp difference between the short and long spacers would provide only a short time (∼2 s) to establish a 5-fold difference in dislodgement capability. If the promoter proximity effect can be confirmed, experiments with a larger range of distances, better controlled stall-site sequences and elongation factor mutations could be used to examine this further.

The wild-type system has two main behavioral regimes. At low RNAP flux, where *k_F_* « *k_T_*+*k_U_*+*k_SD_*, each RNAP almost always acts alone, either terminating or passing through the roadblock site without cooperation from a trailing RNAP. At higher RNAP flux levels, the wild-type system enters a more complex regime, where interactions between multiple RNAPs at the roadblock site provide significant cooperation. In our LacI/*Oid-O1-O2* system, the low-flux regime applies for promoter firing rates of less than 1 every 30 s and the high-flux regime applies for firing rates >1 every 20 s, with an intermediate transition zone. For weaker binding roadblocker proteins, increased *k_U_* and likely increased *k*_SD_ means that *k_F_* « *k_T_*+*k_U_*+*k_SD_* can hold for considerably stronger promoters, extending the low-flux regime.

The critical variables in the low RNAP flux regime are *k_U_* and *k*_SD_, since *k_T_* is likely to be reasonably constant in wild-type cells (Figure 1D). Although we have examined a 23-fold range of *k_U_*, associated with an estimated 4-fold range of *k*_SD_ (Supplementary Figure S2), *k_U_* and *k*_SD_ are likely to fall outside these ranges for some DNA-binding protein/operator combinations. Can we predict the level of roadblocking in these cases? Higher unbinding rates should lead to increased readthrough, and we can calculate using the analytical model (Figure 1D; at 250 nM LacI without any increase in *k*_SD_) that increasing *k_U_* 10-fold beyond that for LacI-*O2* (to *k_U_* ∼0.1 s^−1^) should increase *Rf* from 26% to 68%, while a 100-fold increase in *k_U_* (to *k_U_* = 1.0 s^−1^) would almost eliminate roadblocking (*Rf* = 98%). A 100-fold increase in *k_U_* would give an affinity comparable to the weak *lacO3* operator (43). Allowing *k*_SD_ to increase with increasing *k_U_* does not affect the calculated roadblocking substantially. Using a power law to extrapolate how *k*_SD_ changes with *k_U_* (Supplementary Figure S9A) gave a ∼3-fold increase in *k*_SD_ for a 10-fold increase in *k_U_* and a calculated *Rf* = 72% (at 250 nM LacI), only slightly above the 68% without an increase in *k*_SD_. Thus, once unbinding is fast, dislodgement becomes unimportant.

In the high RNAP flux regime, most readthrough relies on roadblock dislodgement by multiple RNAPs, making *k*_MD_ the critical parameter. We estimate *k*_MD_ to be ∼4- to 10-fold higher than *k*_SD_ if all stalled RNAPs are subject to the same termination rate, and 2- to 4-fold higher than *k*_SD_ if RNAPs can protect each other from termination (this difference depends on operator strength; Supplementary Figure S2). Frequent dislodgement and high flux can make rebinding of the roadblock protein limiting, so that *k_B_* also becomes significant in this regime. For weaker DNA-binding proteins, spontaneous unbinding can also be significant. Increasing *k_U_* to 0.1 s^−1^ would increase λ*pL* readthrough from *Rf* = 45% (for *O2*) to *Rf* = 73% (at 250 nM LacI). If *k*_MD_ values continue to rise with increasing *k_U_* (Supplementary Figure S9A), then readthrough increases further; to *Rf* = 91% for λ*pL* (if *k*_MD_ = 0.1 s^−1^).

### Mechanisms of dislodgement

Our data and literature estimates of the LacI *in vivo* on-rate (40) and promoter firing rates (44) are consistent with a single RNAP being able to actively dislodge a LacI roadblock, but further confirmatory experiments are required. Any dislodgement was fairly slow, taking on average at least 18 min for *Oid* or 4 min for *O2*. This equates to making dissociation of LacI up to ∼7-fold faster from *Oid*, or 1.2-fold faster from *O2*. We imagine that dislodgement may require specific unlikely combinations of microstates of the ternary elongation complex, the LacI–DNA complex and even the intervening DNA; *k*_SD_ is a coarse sum over a large variety of molecular events.

Our analysis indicates that multiple RNAPs also actively increase the dislodgement of the roadblock, as opposed to acting solely by protecting each other from termination. Two non-exclusive basic mechanisms might account for increased dislodgement. (i) In the presence of RNAP restart factors, the leading RNAP can make multiple attempts to transcribe into the roadblock, with the overall success of dislodgement proportional to the number of attempts. In this mechanism, the trailing RNAP aids dislodgement by acting as a restart factor by pushing a backtracked leading RNAP forward (5). (ii) The presence of the trailing RNAP may change the nature (not just the frequency) of dislodgement attempts, somehow applying the energy of additional NTP cleavages to provide a larger or more sustained force against the roadblock–DNA complex (48). The relationship between *k*_SD_ and *k*_MD_ at the three different *k_U_* values we measured (Supplementary Figure S9) suggests that the first model cannot be the sole explanation of cooperation. If trailing RNAPs simply increase the frequency of dislodgement attempts by the leading RNAP, then the ratio *k*_MD_/*k*_SD_ should be the same for the three different *lac* operators. Instead, *k*_SD_ is more sensitive to increased binding affinity than *k*_MD_; in both the standard and the termination protection models, *k*_SD_ drops ∼4.2-fold from *O2* to *Oid*, while *k*_MD_ drops only ∼1.7-fold (Supplementary Figures S2 and S9). A worse fit to the data was obtained if the *k*_MD_/*k*_SD_ ratio was held fixed across all three operators. This suggests that some extra dislodgement capability is available with multiple RNAPs, supporting the second ‘combined push’ model.

### Avoiding excessive roadblocking *in vivo*

It has been proposed that RNAP cooperation is the primary cellular mechanism for preventing excessive loss of transcription due to roadblocking (5,20). However, we find that cooperation is only significant for strong promoters and does not eliminate roadblocking (Figure 5C and D). Even with the very strong λ*pL* promoter, giving a flux of ∼1 RNAP per 5 s, roadblocking by LacI caused loss of 75% to 25% of the transcribing RNAPs, depending on the binding site and LacI concentration. Maximizing cooperation by lowering the termination rate also did not eliminate roadblocking because expression from stronger promoters became limited by promoter clogging (Δ*mfd*; Figure 5C).

**Figure 7.**
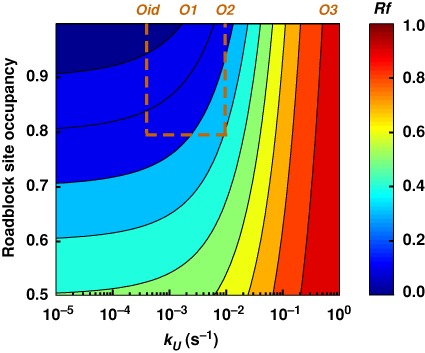
Tuning roadblocking. The roadblocking effect of a protein binding site can be reduced while maintaining its occupancy by increasing both *k_U_* and *k_B_* (keeping *k_B_* /(*k_U_*+*k_B_*) constant). The analytical equation for low RNAP flux (Figure 1D) was used with *k_F_* = 0, *k_T_* = 0.063 and the power law extrapolations for *k*_SD_ versus *k_U_* (see Supplementary Figure S9A). The boxed area shows the parameter region we examined experimentally.

For avoiding deleterious roadblocking, low binding strengths (high *k_U_*) of DNA binding proteins, such that spontaneous unbinding and dislodgement is relatively fast (see above), is a more effective and general mechanism than RNAP cooperation, reducing roadblocking of both weak and strong promoters. However, without compensatory mechanisms, increasing the unbinding rate of a DNA-binding protein from a particular site in order to avoid roadblocking would reduce the site occupancy and may compromise its normal function. Our analysis shows that roadblocking and site occupancy are, to some degree, independently tunable (Figure 7). This is because the other factor affecting site occupancy—the rate of binding of the protein (*k_B_*)—has little effect on roadblocking unless the promoter is very strong (Figure 3).

A high *k_B_* can be achieved with high cellular concentrations of the DNA-binding protein in order to maintain site occupancy. Alternatively, the binding rate can be increased by increasing the on-rate constant, for example, by mechanisms such as facilitated diffusion (49), or increasing the ‘local’ concentration of the roadblocker at the site by cooperative DNA binding. Cooperativity allows use of multiple weak binding sites that each have high individual unbinding rates and thus provide little resistance to RNAP but the cooperative interactions mean that once one of the sites is bound, binding to the other sites is rapid. Reduction of roadblocking may thus be one selective advantage for evolution of these mechanisms.

### Using roadblocking for regulation

On the other hand, what does our analysis tell us about how roadblocking could be maximized for regulatory purposes both in natural and engineered systems? The maximum roadblocking effect we saw was a 25-fold inhibition of transcription from weak to moderate strength promoters when the strongest roadblocker affinity was used (LacI at *Oid*). Whether stronger protein–DNA interactions could reduce dislodgement further to increase roadblock regulation is not clear. Thus, to give a regulatory range comparable to promoter repression, multiple roadblocks may be required.

High levels of roadblocking are seen with the CRISPR system. An up to 35-fold inhibition was seen with an enzymatically inactive dCas9–crRNA complex targeted to DNA ∼250 bp downstream of the promoter (15), while up to 300-fold effects were seen using a dCas9–sgRNA roadblock (16). Increased affinity due to combined protein–DNA and RNA–DNA interactions may explain such high levels of roadblocking, however we suspect that some additional effect may be involved.

An advantage of roadblocking as a regulatory mechanism is that it can be relatively easily added to existing promoter-focused regulation, since a roadblocking site can function well downstream of the promoter. If the regulatory range of the promoter activity is within the low RNAP flux regime, then the roadblock acts rather like a resistor, exerting a constant fold reduction in transcription but still allowing normal regulation to occur. Roadblock regulation of very strong promoters is more complex and is reduced by RNAP cooperation. The maximal inhibition for λ*pL* with a promoter–roadblock spacing of 102 bp was only 4-fold, and that required the highest LacI concentration. However, we achieved 10-fold inhibition of λ*pL* by taking advantage of promoter clogging and preventing cooperation by placing the roadblock site close to the promoter (Figure 6).

Roadblocking could provide extra ultrasensitivity in transcriptional regulation, an effect suggested for RNAP cooperation at strong pause sites (50). If the regulator changes the promoter activity such that the degree of RNAP cooperation changes significantly, for example from the low flux regime to the high flux regime (or vice versa), then the roadblock should magnify the effect of the regulator.

Whether roadblocking could be used for gene regulation in eukaryotic cells is not clear, as there are no examples of transcriptional regulation by endogenous eukaryotic roadblocking proteins. In the presence of LacI, a *lacOid* operator placed in an intron ∼500 bp downstream of the SV40 promoter in rabbit cells caused an unquantified but clearly reduced T-antigen expression, though regulatory mechanisms apart from roadblock were not excluded (13). A dCas9–sgRNA complex targeted downstream of the SV40 promoter gave only 3-fold regulation in human cells (16). Although roadblock encounters may contribute to the substantial *in vivo* pausing of RNAP seen in eukaryotic cells (24), it is not clear that there are mechanisms to efficiently terminate paused transcription. The Mfd-like CSB protein does not appear to have termination activity (51).

### Other factors affecting transcriptional roadblocking

Other factors beyond those we tested are likely to be important for roadblocking. The DNA sequence upstream of the roadblock could affect RNAP–roadblock collisions, RNAP termination probabilities and the probabilities of backtracking and restart (9,23,52). Also, the promoter sequence may have an effect beyond specification of the firing rate through non-random promoter firing, which can produce bunching of elongating RNAPs (53). Promoter bursting should increase cooperation. *In vivo* RNAP elongation factors such as GreA/B (TFIIS), NusG, pppGp(p) and DksA (54) are likely to play important and potentially variable roles depending on growth conditions and roadblock specifics. The roadblock-suppressing effects of trailing ribosomes (33) are also likely to interact with the RNAP flux and roadblocker protein kinetics.

It is also conceivable that dislodgement rates could be different for protein–DNA complexes with the same overall unbinding rate, possibly due to different arrangements of strong and weak atomic level protein–DNA contacts, supported by the strong effect of orientation on roadblocking by dCas9–RNA complexes (15,16). Further systematic, quantitative analyses will be needed to disentangle the effects of these factors on roadblocking.

## SUPPLEMENTARY DATA

Supplementary Data are available at NAR Online.

SUPPLEMENTARY DATA

## References

[1] Shearwin K.E., Callen B.P., Egan J.B. (2005). Transcriptional interference—a crash course. Trends Genet..

[2] McGlynn P., Savery N.J., Dillingham M.S. (2012). The conflict between DNA replication and transcription. Mol. Microbiol..

[3] Helmrich A., Ballarino M., Tora L. (2011). Collisions between replication and transcription complexes cause common fragile site instability at the longest human genes. Mol. Cell.

[4] Deuschle U., Gentz R., Bujard H. (1986). lac repressor blocks transcribing RNA polymerase and terminates transcription. Proc. Natl. Acad. Sci. U.S.A..

[5] Epshtein V., Toulme F., Rahmouni A.R., Borukhov S., Nudler E. (2003). Transcription through the roadblocks: the role of RNA polymerase cooperation. EMBO J..

[6] Sellitti M.A., Pavco P.A., Steege D.A. (1987). lac repressor blocks in vivo transcription of lac control region DNA. Proc. Natl. Acad. Sci. U.S.A..

[7] Chintakayala K., Singh S.S., Rossiter A.E., Shahapure R., Dame R.T., Grainger D.C. (2013). E. coli Fis protein insulates the cbpA gene from uncontrolled transcription. PLoS Genet..

[8] He B., Zalkin H. (1992). Repression of Escherichia coli purB is by a transcriptional roadblock mechanism. J. Bacteriol..

[9] Lewis D.E., Komissarova N., Le P., Kashlev M., Adhya S. (2008). DNA sequences in gal operon override transcription elongation blocks. J. Mol. Biol..

[10] Zalieckas J.M., Wray L.V., Ferson A.E., Fisher S.H. (1998). Transcription-repair coupling factor is involved in carbon catabolite repression of the Bacillus subtilis hut and gnt operons. Mol. Microbiol..

[11] Choi S.K., Saier M.H. (2005). Regulation of sigL expression by the catabolite control protein CcpA involves a roadblock mechanism in Bacillus subtilis: potential connection between carbon and nitrogen metabolism. J. Bacteriol..

[12] Belitsky B.R., Sonenshein A.L. (2011). Roadblock repression of transcription by Bacillus subtilis CodY. J. Mol. Biol..

[13] Deuschle U., Hipskind R.A., Bujard H. (1990). RNA polymerase II transcription blocked by Escherichia coli lac repressor. Science.

[14] Kuhn A., Bartsch I., Grummt I. (1990). Specific interaction of the murine transcription termination factor TTF I with class-I RNA polymerases. Nature.

[15] Bikard D., Jiang W., Samai P., Hochschild A., Zhang F., Marraffini L.A. (2013). Programmable repression and activation of bacterial gene expression using an engineered CRISPR-Cas system. Nucleic Acids Res..

[16] Qi L.S., Larson M.H., Gilbert L.A., Doudna J.A., Weissman J.S., Arkin A.P., Lim W.A. (2013). Repurposing CRISPR as an RNA-guided platform for sequence-specific control of gene expression. Cell.

[17] Neufing P.J., Shearwin K.E., Egan J.B. (2001). Establishing lysogenic transcription in the temperate coliphage 186. J. Bacteriol..

[18] Dodd I.B., Egan J.B. (2002). Action at a distance in CI repressor regulation of the bacteriophage 186 genetic switch. Mol. Microbiol..

[19] Bondarenko V.A., Steele L.M., Ujvari A., Gaykalova D.A., Kulaeva O.I., Polikanov Y.S., Luse D.S., Studitsky V.M. (2006). Nucleosomes can form a polar barrier to transcript elongation by RNA polymerase II. Mol. Cell.

[20] Epshtein V., Nudler E. (2003). Cooperation between RNA polymerase molecules in transcription elongation. Science.

[21] Pavco P.A., Steege D.A. (1990). Elongation by Escherichia coli RNA polymerase is blocked in vitro by a site-specific DNA binding protein. J. Biol. Chem..

[22] Sancar A., Sancar G.B., Rupp W.D., Little J.W., Mount D.W. (1982). LexA protein inhibits transcription of the E. coli uvrA gene in vitro. Nature.

[23] Toulme F., Mosrin-Huaman C., Artsimovitch I., Rahmouni A.R. (2005). Transcriptional pausing in vivo: a nascent RNA hairpin restricts lateral movements of RNA polymerase in both forward and reverse directions. J. Mol. Biol..

[24] Churchman L.S., Weissman J.S. (2011). Nascent transcript sequencing visualizes transcription at nucleotide resolution. Nature.

[25] Nudler E. (2012). RNA polymerase backtracking in gene regulation and genome instability. Cell.

[26] Hodges C., Bintu L., Lubkowska L., Kashlev M., Bustamante C. (2009). Nucleosomal fluctuations govern the transcription dynamics of RNA polymerase II. Science.

[27] Borukhov S., Lee J., Laptenko O. (2005). Bacterial transcription elongation factors: new insights into molecular mechanism of action. Mol. Microbiol..

[28] Toulme F., Mosrin-Hauman C., Sparkowski J., Das A., Leng M., Rahmouni A.R. (2000). GreA and GreB proteins revive backtracked RNA polymerase in vivo by promoting transcript trimming. EMBO J..

[29] Selby C.P., Sancar A. (1995). Structure and function of transcription-repair coupling factor. II. Catalytic properties. J. Biol. Chem..

[30] Park J.S., Marr M.T., Roberts J.W. (2002). E. coli transcription repair coupling factor (Mfd protein) rescues arrested complexes by promoting forward translocation. Cell.

[31] Chambers A.L., Smith A.J., Savery N.J. (2003). A DNA translocation motif in the bacterial transcription–repair coupling factor, Mfd. Nucleic Acids Res..

[32] Dutta D., Chalissery J., Sen R. (2008). Transcription termination factor rho prefers catalytically active elongation complexes for releasing RNA. J. Biol. Chem..

[33] Proshkin S., Rahmouni A.R., Mironov A., Nudler E. (2010). Cooperation between translating ribosomes and RNA polymerase in transcription elongation. Science.

[34] Washburn R.S., Wang Y., Gottesman M.E. (2003). Role of E. coli transcription-repair coupling factor Mfd in Nun-mediated transcription termination. J. Mol. Biol..

[35] Priest D.G., Cui L., Kumar S., Dunlap D.D., Dodd I.B., Shearwin K.E. (2014). Quantitation of the DNA tethering effect in long-range DNA looping in vivo and in vitro using the Lac and lambda repressors. Proc. Natl. Acad. Sci. U.S.A..

[36] Sneppen K., Dodd I.B., Shearwin K.E., Palmer A.C., Schubert R.A., Callen B.P., Egan J.B. (2005). A mathematical model for transcriptional interference by RNA polymerase traffic in Escherichia coli. J. Mol. Biol..

[37] Klumpp S., Hwa T. (2008). Stochasticity and traffic jams in the transcription of ribosomal RNA: intriguing role of termination and antitermination. Proc. Natl. Acad. Sci. U.S.A..

[38] Fange D., Mellenius H., Dennis P.P., Ehrenberg M. (2014). Thermodynamic modeling of variations in the rate of RNA chain elongation of E. coli rrn operons. Biophys. J..

[39] Vassylyev D.G., Vassylyeva M.N., Perederina A., Tahirov T.H., Artsimovitch I. (2007). Structural basis for transcription elongation by bacterial RNA polymerase. Nature.

[40] Elf J., Li G.W., Xie X.S. (2007). Probing transcription factor dynamics at the single-molecule level in a living cell. Science.

[41] Hammar P., Leroy P., Mahmutovic A., Marklund E.G., Berg O.G., Elf J. (2012). The lac repressor displays facilitated diffusion in living cells. Science.

[42] Li G.W., Berg O.G., Elf J. (2009). Effects of macromolecular crowding and DNA looping on gene regulation kinetics. Nat. Phys..

[43] Garcia H.G., Phillips R. (2011). Quantitative dissection of the simple repression input-output function. Proc. Natl. Acad. Sci. U.S.A..

[44] Liang S., Bipatnath M., Xu Y., Chen S., Dennis P., Ehrenberg M., Bremer H. (1999). Activities of constitutive promoters in Escherichia coli. J. Mol. Biol..

[45] Kapanidis A.N., Margeat E., Laurence T.A., Doose S., Ho S.O., Mukhopadhyay J., Kortkhonjia E., Mekler V., Ebright R.H., Weiss S. (2005). Retention of transcription initiation factor sigma70 in transcription elongation: single-molecule analysis. Mol. Cell.

[46] Raffaelle M., Kanin E.I., Vogt J., Burgess R.R., Ansari A.Z. (2005). Holoenzyme switching and stochastic release of sigma factors from RNA polymerase in vivo. Mol. Cell.

[47] Mooney R.A., Davis S.E., Peters J.M., Rowland J.L., Ansari A.Z., Landick R. (2009). Regulator trafficking on bacterial transcription units in vivo. Mol. Cell.

[48] Galburt E.A., Parrondo J.M., Grill S.W. (2011). RNA polymerase pushing. Biophys. Chem..

[49] Tafvizi A., Mirny L.A., van Oijen A.M. (2011). Dancing on DNA: kinetic aspects of search processes on DNA. Chemphyschem.

[50] Klumpp S. (2011). Pausing and backtracking in transcription under dense traffic conditions. J. Stat. Phys..

[51] Selby C.P., Sancar A. (1997). Human transcription-repair coupling factor CSB/ERCC6 is a DNA-stimulated ATPase but is not a helicase and does not disrupt the ternary transcription complex of stalled RNA polymerase II. J. Biol. Chem..

[52] Mosrin-Huaman C., Turnbough C.L., Rahmouni A.R. (2004). Translocation of Escherichia coli RNA polymerase against a protein roadblock in vivo highlights a passive sliding mechanism for transcript elongation. Mol. Microbiol..

[53] Sanchez A., Golding I. (2013). Genetic determinants and cellular constraints in noisy gene expression. Science.

[54] Tehranchi A.K., Blankschien M.D., Zhang Y., Halliday J.A., Srivatsan A., Peng J., Herman C., Wang J.D. (2010). The transcription factor DksA prevents conflicts between DNA replication and transcription machinery. Cell.

