# Supplementary material for: Road rules for traffic on DNA—systematic analysis of transcriptional roadblocking in vivo
Source: Nucleic Acids Res. 2014 Jul 17;42(14):8861–72. doi: 10.1093/nar/gku627 (PMC4132739; doi:10.1093/nar/gku627)
Supplement: SUPPLEMENTARY DATA [file supp_42_14_8861__index.html]

Road rules for traffic on DNA—systematic analysis of transcriptional roadblocking in vivo — SUPPLEMENTARY DATA 

# Road rules for traffic on DNA—systematic analysis of transcriptional roadblocking *in vivo*

## SUPPLEMENTARY DATA

**Files in this Data Supplement:**

- SUPPLEMENTARY DATA
