# Supplementary material for: Road rules for traffic on DNA—systematic analysis of transcriptional roadblocking in vivo
Source: Nucleic Acids Res. 2014 Jul 17;42(14):8861–72. doi: 10.1093/nar/gku627 (PMC4132739; doi:10.1093/nar/gku627)
Supplement: SUPPLEMENTARY DATA [file supp_gku627_nar-01308-z-2014-File009.pdf]

Road rules for traffic on DNA – Systematic analysis of transcriptional roadblocking in vivo

Nan Hao, Sandeep Krishna, Alexandra Ahlgren-Berg, Erin E. Cutts, Keith E. Shearwin, and Ian B. Dodd

Supplementary Methods

Reporter Constructs The parental strain for all reporter assays was *E. coli* strain E4643, a derivative of BW30270 (MG1655 *rph*+) that has had the *lacIZYA* (EcoCyc MG1655: 360,527–366,797) region removed by recombineering (1).

The Δ*mfd* deletion was identical to that of the Keio collection (2), in which the majority of the *mfd* open reading frame was first replaced by a FRT-flanked Kan^R cassette, and subsequently removed by FLP recombinase, leaving a 34-residue scar peptide.

DNA constructions used commercial DNA synthesis (gBlocks from Integrated DNA Technologies; primers from Geneworks), restriction enzyme-based cloning and isothermal Gibson assembly (3).

All *lacZ* reporters were derived from pIT3-CL.*lacZ* (Figure S1A), an integratable plasmid developed from the CRIM plasmids (4), the pZ plasmids (5), and a *lacO2*$^-$ *lacZ* reporter gene (6) preceded by an RNaseIII cleavage site (7). All reporters were integrated into the host chromosome at the λ *attB* site. PCR was used to screen for correct single copy integrants.

The promoter library module (Figure S1A) included 3 natural promoters *pC* (-50 to +40 from phage P2), *pL* (-73 to +90 from phage λ), *pBla* (-171 to +9 from plasmid pBR322) and 26 synthetic promoters. The synthetic promoters were derived either from *pC*, the *pR* promoter of phage 186 (-50 to +9), or the *E. coli* σ^{70} –10/–35 consensus. In each case, a degenerate 17 bp spacer sequence between –10 and –35 was used to create a diversity of promoter strengths (8). The synthetic promoters were selected from a combined screen of ~600 colonies. Once a desired LacZ expression in the absence of LacI was selected, the promoters were sequenced (Table S1). To test the effect of reduced spacing on transcriptional roadblocking, two additional spacers were created. Both the 60bp and 30bp

spacers were derived from 102 bp spacer construct by truncating the 102bp spacer from the +1 position end, leaving the *lac* operator proximal sequence intact (Figure S1A).

The *lac* operator module includes *lacOid*, *lacO1*, or *lacO2* (Figure S1A).

Background LacZ activity from the reporter was measured in each set of assays by using a ‘promoterless’ construct, in which the weakest promoter variant of pIT3-CL.P2*pC*.102.*Oid*.*lacZ* was modified by mutation of 4 positions of the –10 hexamer (Table S1). This ‘promoterless’ reporter gave the same LacZ activity as a reporter that lacked the entire promoter sequence (Figure S1B). A slight sensitivity to LacI remained (Figure S1B), thus two different background values, $bkg_{(-LacI)}$ and $bkg_{(+LacI)}$, were measured in each assay set.

To improve assays of strong promoters (see below), we created a reporter variant, pIT3-CL. *lacZ** in which the native ribosome binding sequence (RBS) of *lacZ* was weakened. The relative strength of the native *lacZ* RBS was predicted using RBS Calculator available at http://www.salis.psu.edu/RBS_Calculator.shtml (9). Reverse engineering was performed with the aim of reducing the strength of the RBS by ~10 fold to assay the strength of very strong promoters. Simultaneously mutating three nucleotides at +289C→A, +302T→G and +304C→G (the start codon is at +326) reduced RBS strength by ~62 fold (see below).

LacI Expression plasmids Derivatives of plasmid pUHA-1 (obtained from H. Bujard, Heidelberg University, Germany), a p15A origin, kanamycin resistance plasmid carrying the wild-type *lacI* gene under the control of its native p*lacI* promoter were used to supply Lac repressors in the cell. Four variants with reduced LacI expression were obtained by screening after random mutagenesis of the –35 hexanucleotide of p*lacI* in pUHA-1. Promoter sequences for all 5 LacI expression plasmids are available on request. A control pUHA-Δ*lacI* plasmid was generated by deleting the entire *lacI* coding region using two flanking SalI sites. Expression levels of LacI from each of the pUHA plasmid variants, relative to a chromosomally-integrated single-copy wild-type p*lacI*.*lacI* construct, were estimated by repression of a *placUV5.lacO2.lacZ* reporter using the method of Priest et al. (10). These were converted to absolute LacI concentrations (Figure 1D) by using the measurement of 11 LacI tetramers per $placI^{+}.lacI^{+}$ cell and assuming a 1 fL cell volume ([LacI]=18 nM) (11).

Minimal Medium LacZ Assays Microtiter plate-based LacZ assays were carried out as reported (10) with minor variations. Cultures grown in microtitre plates in M9 minimal medium (M9MM) supplemented with 2 mM $MgSO_4$, 0.1 mM $CaCl_2$, 0.01 mM $(NH_4)_2Fe(SO_4)_2$, and

0.4% glycerol. After reaching mid-late log phase, cultures were added to a combined lysis-assay buffer in a microtiter plate well containing the following: 50 μL culture/M9MM (either 20 μL of culture plus 30 μL of M9MM or 50 μL of culture), 150 μL of TZ8 (100 mM Tris·HCl, pH 8.0, 1 mM $MgSO_4$, 10 mM KCl), 40 μL of ONPG (o-nitrophenyl-β-D-galactoside 4 mg/mLin TZ8), 1.9 μL of 2-mercapoethanol, and 0.95 μL of polymyxin B (20 mg/mL; Sigma). Assays were performed in triplicate with independent colonies and repeated on at least three different days (*n*=9).

Promoter strength measurement The promoters in our promoter library span almost 3 orders of magnitude in their strength, and we found that the amount of LacZ expressed by some of the very strong promoters in the reporters with the native *lacZ* RBS exceeded the linear range of our LacZ assay. This is shown in Figure S1C, where 12 of the promoters were assayed in two different reporter constructs, one with the native *lacZ* RBS, and the other with the engineered weak RBS (*lacZ**, see above).

To minimize this saturation effect, we assayed the strong promoters in reporters with the weaker *lacZ** RBS. However, the reporter with the stronger *lacZ* RBS was used to give the sensitivity needed to assay the weak promoters. In order to bring the results obtained from both RBS constructs to the same scale, a mathematical transformation was applied.

We assumed that there was negligible non-linearity in the observed LacZ activities (Ow) of the promoters assayed with the weak RBS and thus that these measurements reflect the true relative promoter strengths. To convert the saturation-affected LacZ activities with the strong RBS (Os) to this Ow scale, we used a rectangular hyperbola equation to fit the data in Figure S1C:

$$Os = \frac{Ow * b}{Ow + a}$$

which rearranges to:

$$Ow = \frac{Os * a}{b - Os}$$

The optimal values for a = 34.32 and b = 2120 were fitted using Prism Graphpad software (R^2=0.96). This puts the relative strengths of the strong RBS/ weak RBS = b/a = 62. Following transformation, a close to linear relationship can be obtained for identical promoters regardless of whether they were assayed in native or weak *lacZ* RBS constructs (Figure S1D).

Figure S1E shows that the fractional readthrough (*Rf*) values using this transformation for LacZ activities from the strong RBS measured with and without LacI were very similar to the *Rf* values obtained for the same promoters measured with the weak RBS. The exception is the λ*pL* promoter, which gave a higher readthrough measurement when assayed with the strong RBS. However, we are less confident on our λ*pL* measurement obtained with strong RBS given the big error bar size, due to the limitation of the assay. Importantly, the transformation causes the *Rf* values for the weak promoters (measured with the strong RBS) to align well with the *Rf* values for the strong promoters (measured with the weak RBS) (Figure S1E). Fifteen representative promoters from the library, including the three non-synthetic promoters, were chosen for further study.

Stochastic Modeling The values for the fixed parameters k_F, k_B and k_U (Figures 1C and S2) were calculated as follows:

Promoter firing frequencies (k_F) were calibrated using the λ*pL* and *pBla* promoters, for which in vivo firing rates have been estimated (12). Under similar growth conditions to ours (a doubling time ~83 mins), *pBla* fired approximately once every 120 s, about 12 times slower than λ*pL* (12). However the identical *pBla* and *pL* promoter fragments in our system showed a 49-fold difference in promoter activity. Given this uncertainty in the firing frequencies of *pBla* and *pL*, we calculated our promoter strengths on *pBla* and λ*pL* separately, and used the average values of these two calibrations to calculate the final firing rates in our modelling. This leads to a firing frequency of one transcript every 4.9 s for λ*pL* and every 240 s for *pBla*.

The binding rates for the five different [LacI] were calculated as $k_B = k_{on}$ x [LacI] s^{-1}. The time required for a single Lac repressor tetramer to find its ideal operator *lacOid* in vivo is ~4 mins (13-15). A single molecule in a 1 fL *E. coli* cell is 1.66 nM, giving an on-rate constant k_{on} = $1/(240 \times 1.66 \times 10^{-9}) = 2.51 \times 10^{6}\ M^{-1} s^{-1}$.

The unbinding rates for LacI from the three *lac* operators (Figure 1D) were calculated from the in vivo dissociation constants K_D – 170 pM, 0.9 nM, and 3.9 nM for *Oid*, *O1* and *O2*, respectively (11) – and the relation $k_U = K_D \times k_{on}$.

The stochastic simulation was implemented with a hybrid-Gillespie method for increased efficiency and reduced complexity. When there is no RNAP on the simulated DNA fragment, only three processes are possible, that is loading of a new RNAP at the promoter, and binding or unbinding of LacI, depending on the current state of the *lac* operator. Using the

Gillespie approach, the next time for each of these processes can be approximated as '–ln(r/k)', where r is a random number between 0 and 1 and k is the rate of the process. The system is updated with the process with the shortest next time, and the time is advanced by the time associated with that process. If the process is the binding or unbinding of the LacI, the procedure is repeated until an RNAP is loaded on the DNA. The simulation then proceeds by fixed time steps until all RNAPs are removed from the DNA and the simulation reverts to the Gillespie approach.

In the fixed time step approach, each step is set to the time taken for RNAP to move forward one base pair; in each time step, each RNAP attempted to advance 1 bp. All other events (see Figure 1C) are assigned a rate k, (= k_F, k_T, k_U, k_B, k_{SD} or k_{MD}) and if a particular event is possible, its occurrence during the next time step is decided by generating a random number between 0 and 1; if this number is less than $1\text{-}e^{-k}$, then that event occurs.

The order of attempted events is as follows:

1. *LacI binding or spontaneous unbinding.* Binding only occurs if none of the operator positions is overlapped by RNAP. Bound LacI occupies all 20bp of the operator site.
2. *RNAP movement.* Starting with the RNAP furthest from the promoter, each RNAP (paused or not) attempts to move forward 1 bp. If the RNAP is blocked either by LacI or by a paused RNAP, it only moves forward if itself or the leading RNAP dislodges the LacI, through application of the k_{SD} or k_{MD} rate (depending on whether there is one or more RNAP stalled at the roadblock). Thus a successful dislodgement will enable all RNAP queued at the roadblock to advance. Any RNAP that is unable to move forward is considered paused. If the back of an RNAP passes the last position of the operator, a new transcript is counted and that RNAP is eliminated from the DNA.
3. *Termination.* Each paused RNAP can be removed from the system with rate k_T.
4. *Promoter firing*. If there is no RNAP overlapping positions +1 to +5 of the DNA (the 1st bp of DNA in the model is at -25), a new RNAP front is loaded at position +5 with rate k_F.

The simulations were continued until 1,000 RNAPs passed the roadblock for weak promoters and 10,000 RNAPs passed the roadblock for strong promoters. *Rf* was calculated as the ratio of the number of RNAPs passing the last base of the operator per unit time in the presence of the roadblocker protein divided by the number passing per unit time in its absence.

Parameter fitting Simulations and parameter fitting were performed on an SGI Altix XE1300 high performance computer hosted by eResearch SA (Adelaide). The three unknown

parameters k_T, k_{SD}, and k_{MD} were fitted with a classic Monte Carlo simulated annealing approach.

For any parameter set, expected *Rf* values were calculated by carrying out simulations for each of the experimentally tested promoters. These were then compared with the experimentally observed *Rf* values by calculating a score=$\Sigma[(Rf_{observed} - Rf_{expected})^2/Rf_{expected}]$. All fitted parameter values were then varied at random within a 2 fold range, and a new score was calculated. If the new score was better than the previous score, the new parameter set was retained, and the score was updated, otherwise the new parameter set was rejected. A typical run involved 100,000 iterations of fitting, which took ~50 hours to run when split into 50 computing nodes.

The program, written in FORTRAN, is available on request.

Parameter sensitivity analysis

We tested the sensitivity of the model to variation in the fixed parameter values:

Length of DNA occupied by elongating RNAP, l = 30 bp.

Measurements of the size of the DNA region protected from nucleases by an RNAP stalled at a roadblock range from 25-30 bp (16), congruent with the 30 bp estimate from crystallography (17). We found that the effect on the model predictions of setting this parameter to 25 bp or 40 bp (while keeping other parameters at their optimal values for l = 30 bp) was small (Figure S5A). Differences are only seen at very high promoter strengths, primarily due to increased promoter clogging as l becomes large enough to reduce the number of RNAPs that can queue at the roadblock.

RNAP elongation speed, v = 40 bp s^{-1}.

Literature measurements of average in vivo RNAP elongation velocity range from 25 to 80bp/s (18-20). The effect on the model predictions of setting this parameter to 25 bp or 60 bp (while keeping other parameters at their optimal values for $v = 40$ *bp* s^{-1}) was not large (Figure S5B). The sensitivity analysis (Figure S6) showed little effect of RNAP speed on our k_T, k_{SD}, and k_{MD} estimates, with a slight tendency for these parameter values to decrease with increasing speed.

Promoter firing rates

To test the sensitivity of the fitted estimates for k_T, k_{SD} and k_{MD} to variation in the *absolute* promoter firing rate to the model, fitting was repeated, allowing k_F for λ*pL* to vary 5-fold up

and down from 1 to 1/25 s^{-1}, and scaling all the other promoters accordingly. Good fits to the data were obtained across this range of k_F calibration, with the optimal fitted values for k_T and k_{MD} scaling roughly linearly with the changes in k_F (Figure S6). In general, faster rates of termination and dislodgement are needed if the promoters are firing faster than our calibration; while slower rates are needed if the promoters are slower. The change in k_{SD} is similar to k_T and k_{MD} if k_F is no lower than 2-fold slower than our estimate, but acceptable k_{SD} values fall more quickly if k_F is slower than this (Figure S6). Note that the promoters must fire ~5-fold slower for k_{SD} to approach zero, while $k_{MD} > 0$ holds over this range.

LacI kinetics

The properties of LacI binding to the operator in our model are determined by three fixed parameters, the dissociation constant for each operator $K_{D,}$ the [LacI], and the on-rate constant k_{on}.

– In vivo K_D values for *Oid*, *O1* and *O2* (Figure S2) were determined by Garcia and Phillips (11) by measuring repression factors at six defined cellular LacI concentrations (tetramers/cell) measured by quantitative Western blotting. Errors in the K_D estimates were ca. ± 20% (0.2 *kT* variation in free energies) (11).

– Our [LacI] estimates are based on our measured repression factors, or the fractional occupation, θ, of *O2* by LacI at the different concentrations (10). θ is given by [LacI]/([LacI] + K_D), where K_D is for *O2*. This rearranges to [LacI] = K_D x θ /(1–θ). Thus the relative [LacI] values for our 5 concentrations can be calculated from their respective θ values, as we can assume K_D is constant. The errors for θ were ca. ± 7%. To place these relative concentrations on an absolute scale, we calibrated them relative to the θ we observed for LacI produced from a single-copy *lacI* gene, for which we used the measurement of 11 ± 2 tetramers per cell from Garcia and Phillips (11). The *O2* K_D obtained from this calibration was 3.7 nM, very similar to the 3.9 nM obtained by Garcia and Phillips (11). Propagating the 7% error in relative concentrations and the 18% error (2/11) in absolute concentration gives a ca. ± 20% error for our [LacI] values.

– Errors for the k_{on} estimate are not available.

These values were used to derive k_U and k_B that define LacI kinetics in the stochastic algorithm, using the relations $k_U = K_D \times k_{on}$ and k_B = [LacI] x k_{on}, which means that k_U and k_B vary in parallel with variation in k_{on}. If k_{on} is slower than the literature estimate, then k_U and k_B are slower (slow LacI kinetics); if k_{on} is faster than the literature estimate, then k_U and k_B are faster (fast LacI kinetics). The ratio of k_U and k_B is reasonably well fixed by the above estimates, since $k_U/k_B = (1–\theta)/\theta$ (for a given operator and [LacI]), with an error of ± 7% (see above). Thus the primary uncertainty in LacI kinetics stems from the uncertainty in the k_{on}

estimate. We tested the effect of this uncertainty on our fitted estimates for k_T, k_{SD} and k_{MD} by varying k_U over a 100-fold range while holding k_U/k_B constant (i.e. changing k_U *and* k_B in parallel). We found that reasonable fits to the *Oid*/250 nM LacI data were possible over this range. The k_T and k_{MD} estimates were fairly insensitive to k_U over this range, changing only ~2-fold and ~4-fold, respectively. The k_{SD} estimate is also weakly sensitive to lower values of k_U but is strongly affected once k_U reaches values ~6-fold higher than our estimate, with much lower values for k_{SD} becoming possible. Effectively, fast LacI kinetics allow readthrough by spontaneous LacI unbinding (the escape mechanism) rather than requiring a single RNAP to actively dislodge LacI.

Supplementary References

1. Cui, L., Murchland, I., Shearwin, K.E. and Dodd, I.B. (2013) Enhancer-like long-range transcriptional activation by lambda CI-mediated DNA looping. *Proc Natl Acad Sci U S A*, **110**, 2922-2927.

2. Baba, T., Ara, T., Hasegawa, M., Takai, Y., Okumura, Y., Baba, M., Datsenko, K.A., Tomita, M., Wanner, B.L. and Mori, H. (2006) Construction of Escherichia coli K-12 in-frame, single-gene knockout mutants: the Keio collection. *Mol Syst Biol*, **2**, 2006 0008.

3. Gibson, D.G., Young, L., Chuang, R.Y., Venter, J.C., Hutchison, C.A., 3rd and Smith, H.O. (2009) Enzymatic assembly of DNA molecules up to several hundred kilobases. *Nat Methods*, **6**, 343-345.

4. Haldimann, A. and Wanner, B.L. (2001) Conditional-replication, integration, excision, and retrieval plasmid-host systems for gene structure-function studies of bacteria. *J Bacteriol*, **183**, 6384-6393.

5. Lutz, R. and Bujard, H. (1997) Independent and tight regulation of transcriptional units in Escherichia coli via the LacR/O, the TetR/O and AraC/I1-I2 regulatory elements. *Nucleic Acids Res*, **25**, 1203-1210.

6. Muller, J., Oehler, S. and Muller-Hill, B. (1996) Repression of lac promoter as a function of distance, phase and quality of an auxiliary lac operator. *Journal of molecular biology*, **257**, 21-29.

7. Linn, T. and St Pierre, R. (1990) Improved vector system for constructing transcriptional fusions that ensures independent translation of lacZ. *J Bacteriol*, **172**, 1077-1084.

8. Solem, C. and Jensen, P.R. (2002) Modulation of gene expression made easy. *Appl Environ Microbiol*, **68**, 2397-2403.

9. Salis, H.M., Mirsky, E.A. and Voigt, C.A. (2009) Automated design of synthetic ribosome binding sites to control protein expression. *Nat Biotechnol*, **27**, 946-950.

10. Priest, D.G., Cui, L., Kumar, S., Dunlap, D.D., Dodd, I.B. and Shearwin, K.E. (2014) Quantitation of the DNA tethering effect in long-range DNA looping in vivo and in vitro using the Lac and lambda repressors. *Proc Natl Acad Sci U S A*, **111**, 349-354.

11. Garcia, H.G. and Phillips, R. (2011) Quantitative dissection of the simple repression input-output function. *Proc Natl Acad Sci U S A*, **108**, 12173-12178.

12. Liang, S., Bipatnath, M., Xu, Y., Chen, S., Dennis, P., Ehrenberg, M. and Bremer, H. (1999) Activities of constitutive promoters in Escherichia coli. *Journal of molecular biology*, **292**, 19-37.

13. Elf, J., Li, G.W. and Xie, X.S. (2007) Probing transcription factor dynamics at the single-molecule level in a living cell. *Science*, **316**, 1191-1194.

14. Hammar, P., Leroy, P., Mahmutovic, A., Marklund, E.G., Berg, O.G. and Elf, J. (2012) The lac repressor displays facilitated diffusion in living cells. *Science*, **336**, 1595-1598.

15. Li, G.W., Berg, O.G. and Elf, J. (2009) Effects of macromolecular crowding and DNA looping on gene regulation kinetics. *Nature Physics*, **5**, 294-297.

16. Pavco, P.A. and Steege, D.A. (1990) Elongation by Escherichia coli RNA polymerase is blocked in vitro by a site-specific DNA binding protein. *J Biol Chem*, **265**, 9960-9969.

17. Vassylyev, D.G., Vassylyeva, M.N., Perederina, A., Tahirov, T.H. and Artsimovitch, I. (2007) Structural basis for transcription elongation by bacterial RNA polymerase. *Nature*, **448**, 157-162.

18. Vogel, U. and Jensen, K.F. (1994) The RNA chain elongation rate in Escherichia coli depends on the growth rate. *J Bacteriol*, **176**, 2807-2813.

19. Epshtein, V. and Nudler, E. (2003) Cooperation between RNA polymerase molecules in transcription elongation. *Science*, **300**, 801-805.

20. Kemp, P., Gupta, M. and Molineux, I.J. (2004) Bacteriophage T7 DNA ejection into cells is initiated by an enzyme-like mechanism. *Molecular microbiology*, **53**, 1251-1265.

Table S1. Promoter sequences. Sequence alignment between promoterless, P2*pC*, *pBla*, λ*pL* and 26 synthetic promoters at -50 to +9 with the -10 and -35 region highlighted in red. The estimated *absolute* firing rates (s^{-1}) and promoter strength (s per firing) are also shown. 95% CI, 95% confidence intervals (n=9)

Code	Sequence	Identity	*kF* (s^{-1})	*kF* (95% CI)	Strength (s)	Strength (95% CI)
AH2137	TGTCGTCCTCTAGCCCTCATGTACGCCAAGATACTGGACAGCAGGCCTTTTAGTGCCCA	Promoterless	N/A	N/A	N/A	N/A
AH2117	TGTCGTCCTCTAGCCCTCATGTACGCCAAGATACTGGATATAATGCCTTTTAGTGCCCA	P2*pC* derivative	0.00033	0.00002	3016.31	170.19
AH2116	TGTCGTCCTCTAGCCCTCATGGATTGACCGACTGTTACTATAATGCCTTTTAGTGCCCA	P2*pC* derivative	0.00036	0.00004	2744.00	268.45
AH2123	TGTCGTCCTCTAGCCCTCATGTAGCAGTTCACAGGGTTTATAATGCCTTTTAGTGCCCA	P2*pC* derivative	0.00048	0.00002	2081.89	107.29
AH2126	TGTCGTCCTCTAGCCCTCATGAGAGCTGCTTGAGCGTATATAATGCCTTTTAGTGCCCA	P2*pC* derivative	0.00065	0.00003	1528.36	71.21
AH2125	TGTCGTCCTCTAGCCCTCATGAGGCCAAAAAAGATCGATATAATGCCTTTTAGTGCCCA	P2*pC* derivative	0.00069	0.00003	1447.27	64.59
AH2118	TGTCGTCCTCTAGCCCTCATGAATCGTCACGAACTTTGTATAATGCCTTTTAGTGCCCA	P2*pC* derivative	0.00086	0.00003	1158.26	40.27
AH2101	TGTCGTCCTCTAGCCCTCATGAATTGCGTTTAATGTCTTATAATGCCTTTTAGTGCCCA	P2*pC*	0.00136	0.00004	735.36	20.32
AH2122	TGTCGTCCTCTAGCCCTCATGATGAAAGATGGATGGTGTATAATGCCTTTTAGTGCCCA	P2*pC* derivative	0.00264	0.00007	378.48	10.55
AH2119	TGTCGTCCTCTAGCCCTCATGGTAATCGCTTGGTAATGTATAATGCCTTTTAGTGCCCA	P2*pC* derivative	0.00332	0.00012	300.97	10.84
AH2157	ATTTTTCTAAATACATTCAAATATGTATCCGCTCATGAGACAATAACCCTGATAAATGC	*pBla*	0.00412	0.00015	242.99	8.66
AH2121	TGTCGTCCTCTAGCCCTCATGATTCAGAACGAATAGTGTATAATGCCTTTTAGTGCCCA	P2*pC* derivative	0.00480	0.00022	208.17	9.42
AH2127	TGTCGTCCTCTAGCCCTCATGGACGGATGAGAATATAATATAATGCCTTTTAGTGCCCA	P2*pC* derivative	0.00598	0.00017	167.35	4.67
AH2120	TGTCGTCCTCTAGCCCTCATGTAATCTAATAGTGTATATATAATGCCTTTTAGTGCCCA	P2*pC* derivative	0.00629	0.00014	158.86	3.59
AH2129	TGTCGTCCTCTAGCCCTCATGCATTATGTGTAGTACGCTATAATGCCTTTTAGTGCCCA	P2*pC* derivative	0.00690	0.00026	144.96	5.54
AH2130	TGTCGTCCTCTAGCCCTCATGATGATCGCATTTTATGTTATAATGCCTTTTAGTGCCCA	P2*pC* derivative	0.00710	0.00040	140.87	7.89
AH2124	TTTCGTCCTCTAGCCCTCATGCCCGGACATATGGTACATATAATGCCTTTTAGTGCCCA	P2*pC* derivative	0.00873	0.00025	114.57	3.27
AH2131	TGTCGTCCTCTAGCCCTCATGTCAGAGATATAATAGTATATAATGCCTTTTAGTGCCCA	P2*pC* derivative	0.01031	0.00044	96.95	4.12
AH2128	TGTCGTCCTCTAGCCCTCATGTTATATTAGAAGTATGATATAATGCCTTTTAGTGCCCA	P2*pC* derivative	0.01269	0.00035	78.79	2.18
AH2153	TTTCGATAAAACCTATTTACTGTGACCTAAAGAACGCATATATTTTGGCTAAACCCACG	186*pR* derivative	0.01782	0.00080	56.12	2.52
AH2154	TTTCGATAAAACCTATTTACTATTGGTTATGCTATGCGTATATTTTGGCTAAACCCACG	186*pR* derivative	0.01946	0.00097	51.39	2.57
AH2155	TTTCGATAAAACCTATTTACTCCGAACGATGTTCGGAATATATTTTGGCTAAACCCACG	186*pR* derivative	0.02659	0.00072	37.61	1.03
AH2148	TGTCGTCCTCTAGACTTGACAGACTATACCCCCACTTTTATAATGCCTTTTAGTGCCCA	σ70 consensus derivative	0.03113	0.00307	32.12	3.17
AH2146	TTTCGATAAAACCTATTTACTACCTGGGAAACAGCTAGTATATTTTGGCTAAACCCACG	186*pR* derivative	0.03901	0.00122	25.64	0.80
AH2151	TGTCGTCCTCTAGCCTTGACAGGCCTTTACTCACAGAGTATAATGCCTTTTAGTGCCCA	σ70 consensus derivative	0.07985	0.00196	12.52	0.31
AH2156	TGTCGTCCTCTAGCCTTGACAGCACGCGTGGCAAAAAATATAATGCCTTTTAGTGCCCA	σ70 consensus derivative	0.08866	0.00305	11.28	0.39
AH2149	TGTCGTCCTCTAGCCTTGACAGGGCGACTTAGACTGAGTATAATGCCTTTTAGTGCCCA	σ70 consensus derivative	0.09883	0.00182	10.12	0.19
AH2152	TGTCGTCCTCTAGCCTTGACAGCGTAGTCCGTGTTCAATATAATGCCTTTTAGTGCCCA	σ70 consensus derivative	0.09917	0.00257	10.08	0.26
AH2150	TGTCGTCCTCTAGCCTTGACATGAAACAGTAGAAGCAATATAATGCCTTTTAGTGCCCA	σ70 consensus derivative	0.10001	0.00459	10.00	0.46
AH2145	TTATCTCTGGCGGTGTTGACATAAATACCACTGGCGGTGATACTGAGCACATCAGCAGG	λ*pL*	0.20250	0.00649	4.94	0.16

Figure S1

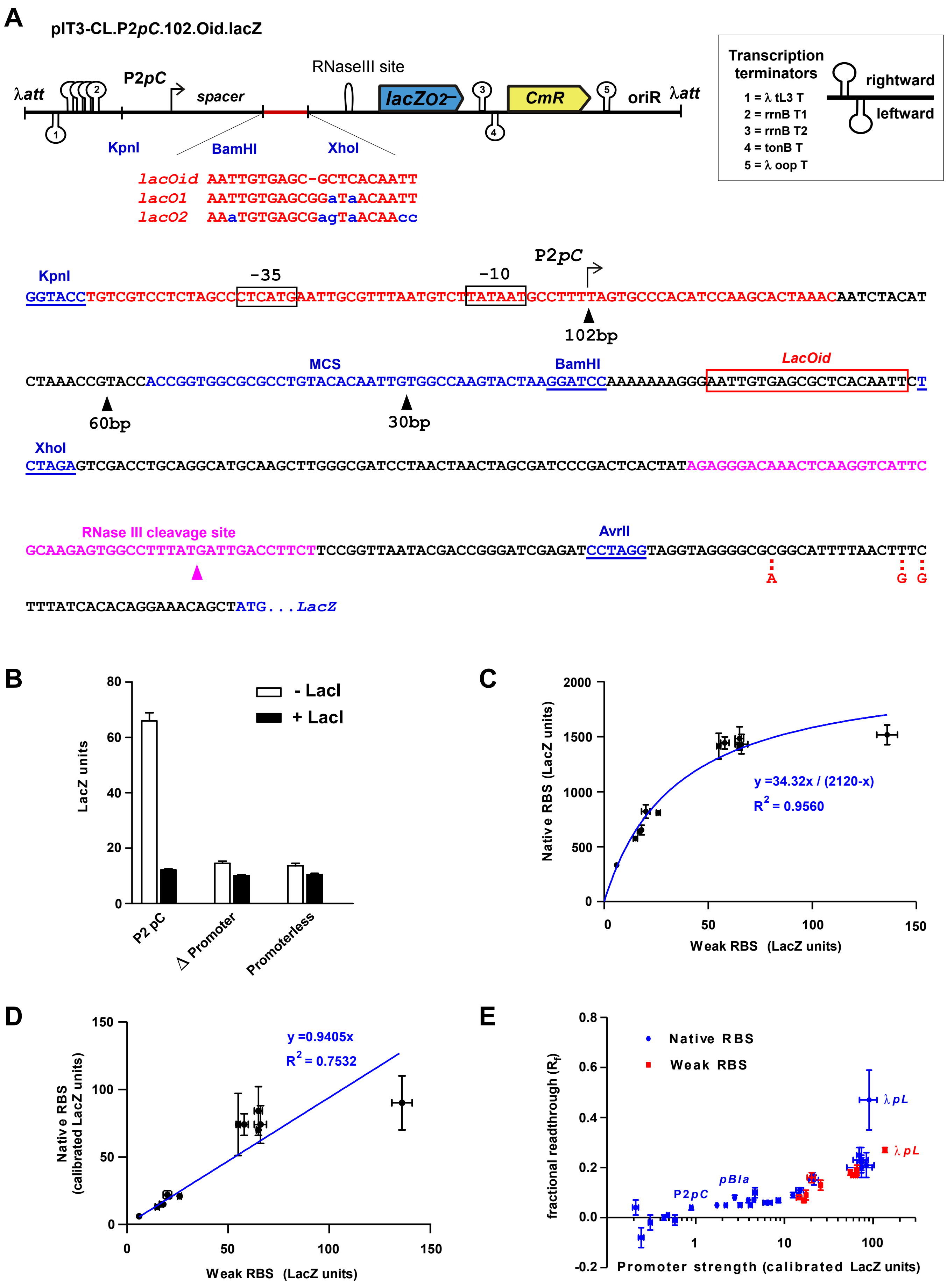
A
pIT3-CL.P2pC.102.Oid.lacZ
λatt
P2pC
spacer
RNaseIII site
lacZO2
CmR
oriR
λatt
KpnI
BamHI
XhoI
lacOid AATTGTGAGC-GCTCACAATT
lacO1 AATTGTGAGCGGaTaACAATT
lacO2 AAaTGTGAGCGagTaACAAcc
Transcription terminators
rightward
leftward
1 = λ tL3 T
2 = rrnB T1
3 = rrnB T2
4 = tonB T
5 = λ oop T
KpnI
-35
-10
P2pC
GGTACCTGTCGTCCTCTAGCCCTCATGAATTGCGTTTAATGTCTTATAATGCCTTTTAGTGCCCACATCCAAGCACTAAACAATCTACAT
102bp
MCS
BamHI
LacOid
CTAAACCGTACCACCGGTGGCGCGCCTGTACACAATTGTGGCCAAGTACTAAGGATCCAAAAAAAGGGAATTGTGAGCGCTCACAATTCT
60bp
30bp
XhoI
CTAGAGTCGACCTGCAGGCATGCAAGCTTGGGCGATCCTAACTAACTAGCGATCCCGACTCACTATAGAGGGACAAACTCAAGGTCATTC
RNase III cleavage site
AvrII
GCAAGAGTGGCCTTTATGATTGACCTTCTTCCGGTTAATACGACCGGGATCGAGATCCTAGGTAGGTAGGGGCGCGGCATTTTAACTTTC
A
G G
TTTATCACACAGGAAACAGCTATG...LacZ
B
LacZ units
- LacI
+ LacI
P2 pC
Δ Promoter
Promoterless
C
Native RBS (LacZ units)
Weak RBS (LacZ units)
y =34.32x / (2120-x)
R2 = 0.9560
D
Native RBS (calibrated LacZ units)
Weak RBS (LacZ units)
y =0.9405x
R2 = 0.7532
E
fractional readthrough (Rf)
Promoter strength (calibrated LacZ units)
Native RBS
Weak RBS
λpL
λpL
pBla
P2pC

Figure S1. Reporter constructs and activities. A) The upper section shows the basic structure of the integrated reporter vector with the three alternative *lac* operator sequences highlighted, together with relevant restriction sites and transcription terminators. The lower section shows the gBlock sequence used for the construction of pIT3-CL.P2*pC*.102.*Oid*.*lacZ* integration reporter up to the start codon of *lacZ*. Restriction sites and MCS (multiple cloning site) are highlighted in blue, P2*pC* promoter sequence (-50 to +40) in red, RNaseIII cleavage site in magenta with the cleavage position marked by a magenta triangle, and the -35, -10, and *lacOid* sequences boxed. Inclusion of an RNaseIII cleavage site ensures constant *lacZ* gene translation independent of upstream sequences. RBS mutations used to reduce LacZ expression (*lacZ**) are shown in red underneath the wild type sequence. 60 bp and 30 bp spacer constructs were generated by truncating the 102bp spacer from the +1 promoter position while leaving the operator proximal sequence intact. The truncation points are indicated by black triangles. *CmR*, chloramphenicol resistance gene; *lacZO2*$^{-}$, *lacZ* gene with the internal *lacO2* site mutated. B) The LacZ units obtained from P2*pC.Oid* roadblock reporter and control reporters with either promoter deletion (Δpromoter) or –10 hexamer mutation (promoterless, Table S1) in the presence or absence of 250nM of LacI. The promoterless construct produces essentially the same LacZ units as the promoter deletion construct, and was thus used as a background control. Error bars are 95% confidence intervals (*n*=9). C) LacZ units obtained from identical promoter pairs expressing *lacZ* with its native RBS or the weak RBS (*lacZ**). D) Rectangular hyperbola transformation (Supplemental Experimental Procedures) reduces the non-linearity in LacZ assays for fast firing promoters. E) Rf values for identical promoter pairs assayed in either native (blue) or engineered weak (red) RBS constructs. Following transformation, very similar *Rf* values were observed for promoters with the same firing rates regardless of whether they were assayed with native or weak RBS constructs.

Figure S2

Parameter	Meaning	Values
**FIXED PARAMETERS**		
l	Length of elongating RNAP (bp)	30
v	Rate of RNAP elongation (bp s^{-1})	40
k_F	Rate of promoter firing (s^{-1})	0.00033 - 0.20
kon	On rate constant for LacI to lacO (M^{-1} s^{-1})	2.51E+06
k_U	Unbinding rates of LacI to (s^{-1})	
	lacOid, *ladO1*, & *lacO2*	0.00043, 0.0023, 0.0098
k_B	Binding rates of LacI to operator (s^{-1})	
	254nM, 119nM, 70nM, 36nM, 15nM	0.65, 0.30, 0.18, 0.091, 0.037

FITTED PARAMETERS

**wt, 102bp spacer, *lacOid*, 254nM LacI (Figures 1 and 2)**		***Standard model***	***Termination protection***	***Hybrid model***
k_T	Rate of RNAP termination (s^{-1})	0.066	0.12	0.087
k_{SP}	Rate of dislodgement by single RNAP (s^{-1})	0.0015	0.0096	0.0025
k_{MP}	Rate of dislodgement by multiple RNAPs (s^{-1})	0.026	0.0096	0.016

**wt, 102bp spacer, global fitting with 3 *lacO* sites and 5 [LacI]**		***Standard model***			***Hybrid model***		
		Oid	***O1***	***O2***	***Oid***	***O1***	***O2***
k_T	Rate of RNAP termination (s^{-1})	0.063	0.063	0.063	0.085	0.085	0.085
k_{SD}	Rate of dislodgement by single RNAP (s^{-1})	0.0027	0.0063	0.012	0.0042	0.0094	0.018
k_{MD}	Rate of dislodgement by multiple RNAPs (s^{-1})	0.028	0.031	0.047	0.018	0.019	0.029

**Δ*mfd* , 102bp spacer, global fitting with 2 *lacO* sites and 3 [LacI]**		***Oid***	***O2***
k_T	Rate of RNAP termination (s^{-1})	0.0045	0.0045
k_{SD}	Rate of dislodgement by single RNAP (s^{-1})	0.0033	0.0054
k_{MD}	Rate of dislodgement by multiple RNAPs (s^{-1})	0.011	0.011

**wt, *lacOid*, 254nM LacI**		***Increased dislodgement model***		***Decreased termination model***	
		60bp	***30bp***	***60bp***	***30bp***
k_T	Rate of RNAP termination (s^{-1})	0.066	0.066	0.049	0.011
k_{SD}	Rate of dislodgement by single RNAP (s^{-1})	0.0031	0.0080	0.0015	0.0015
k_{MD}	Rate of dislodgement by multiple RNAPs (s^{-1})	0.031	0.026	0.026	0.026

Figure S2. Fixed and fitted parameter values. See the main text and the ‘Sensitivity analysis’ section in the Supplementary Methods for calculations and error estimates.

Figure S3

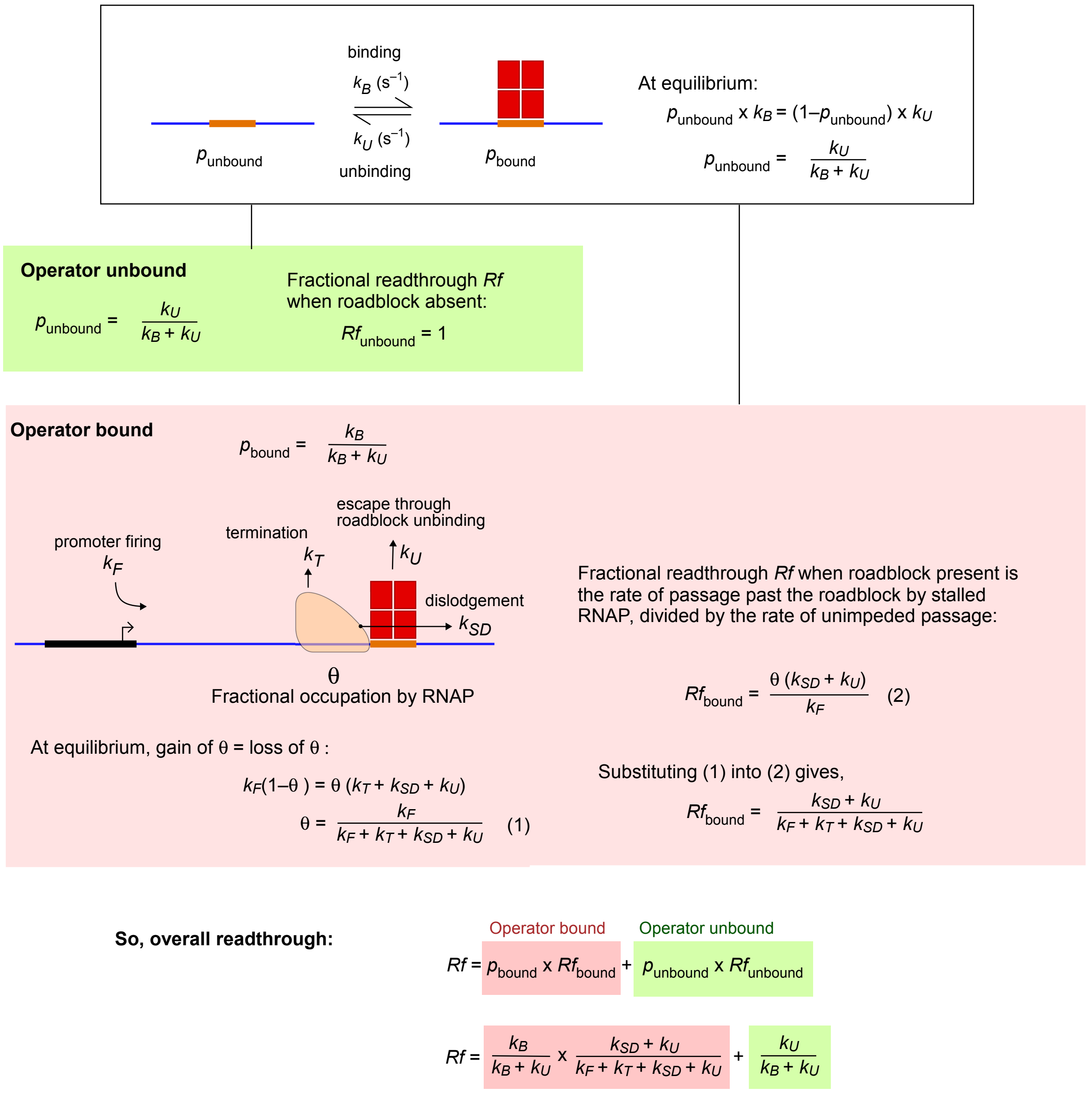

Figure S3. Analytical model. Analytical equilibrium model for readthrough when there is never more than one RNAP stalled at the roadblock. The model applies when $k_F \ll k_T + k_U + k_{SD}$, which holds for weak promoters in this study. The model also makes the assumption that the elongation rate of RNAP is much faster than the LacI binding rate, such that LacI unbinding always allows a stalled RNAP to move into the operator before LacI can rebind. Related to Figure 1.

Figure S4

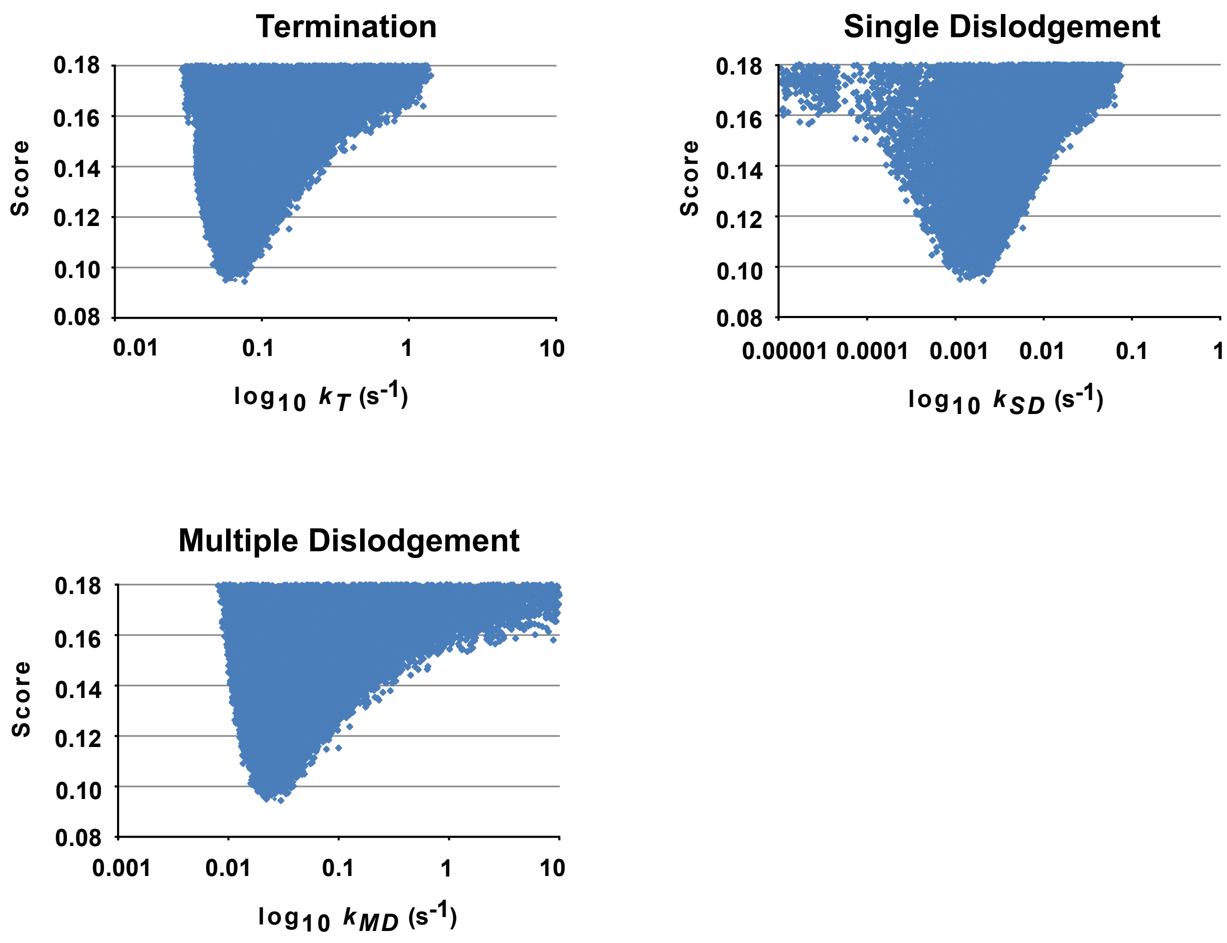

Figure S4. Funnel plot analysis. Parameter fitting was performed for *lacOid* and 250nM LacI (see also Figure 2A) and the parameter value and corresponding fitting score were plotted. The three fitted parameters k_T (A), k_{SD} (B), and k_{MD} (C) converge on clear optimal values.

Figure S5

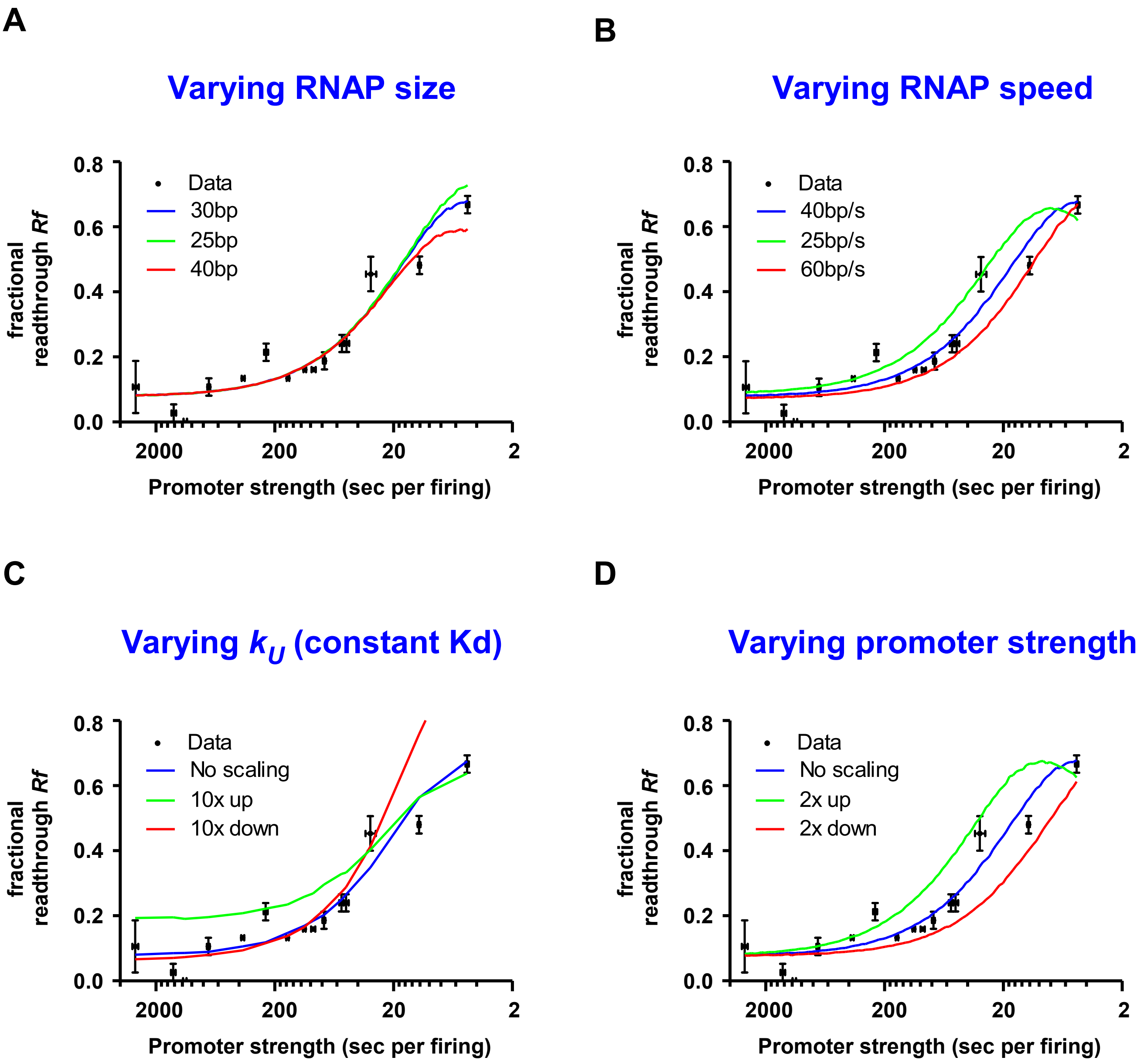

Figure S5. Effects of variation in fixed parameters. The effect on the model predictions for *Oid* at 250 nM LacI of variation in (A) the size of RNAP, *l*, (B) the speed of RNAP, *v*, (C) LacI kinetics (changes in the off-rate k_U were compensated by parallel changes in the binding rate k_B in order to keep the dissociation constant fixed), (D) the firing rates k_F for the promoters (k_F for all promoters changed in parallel). All other parameters were fixed. Sensitivity of the fitted parameters to *v*, k_U, and k_F is shown in Figure S6.

Figure S6

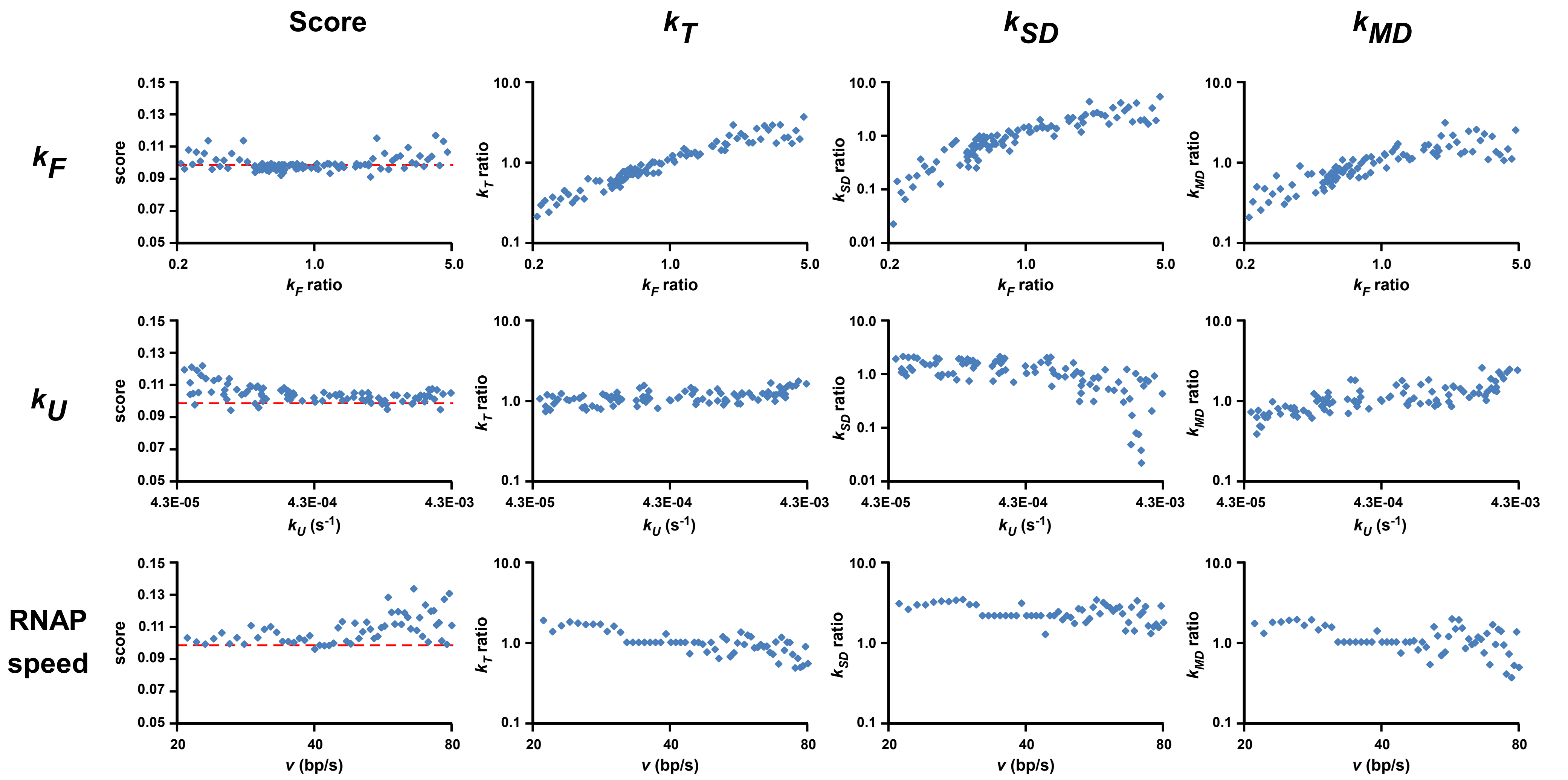

Score
k_T
k_{SD}
k_{MD}
k_F
k_U
RNAP speed
score
k_T ratio
k_{SD} ratio
k_{MD} ratio
0.15
0.13
0.11
0.09
0.07
0.05
10.0
1.0
0.1
0.01
0.2
5.0
k_F ratio
4.3E-05
4.3E-04
4.3E-03
k_U (s^{-1})
20
40
80
v (bp/s)

Figure S6. Sensitivity analysis. The effect of variation in fixed parameters k_F (promoter firing rate), k_U (LacI off-rate) and v (RNAP speed) on fitted values for k_T, k_{SD}, and k_{MD}. Fitting was for *lacOid* at 250 nM LacI for 15 promoters. The range of fixed parameter values (relative to the standard estimate, Figure S2) is given on the x-axes (all other fixed parameter values were held constant). When varying k_F, all 15 promoter values were scaled by the same factor. The fitting score or fold change in the fitted parameter estimate (compared to the standard estimate: k_T, k_{SD}, and k_{MD} = 0.066, 0.0015, and 0.026 s^{-1}, respectively) is given on the y-axes. Each point for the k_U and v tests gives the values for the best fit (lowest score) obtained from 100 fittings. In the k_F tests, the points are the 100 best scoring parameter combinations from 70,000 simulations with random values of k_T, k_{SD}, and k_{MD} chosen over a 100-fold range. Note that for stochastic simulations, the simulated values (and thus the scores) will always vary slightly even for two identical runs. See the ‘Sensitivity analysis’ section in the Supplementary Methods for discussion.

Figure S7

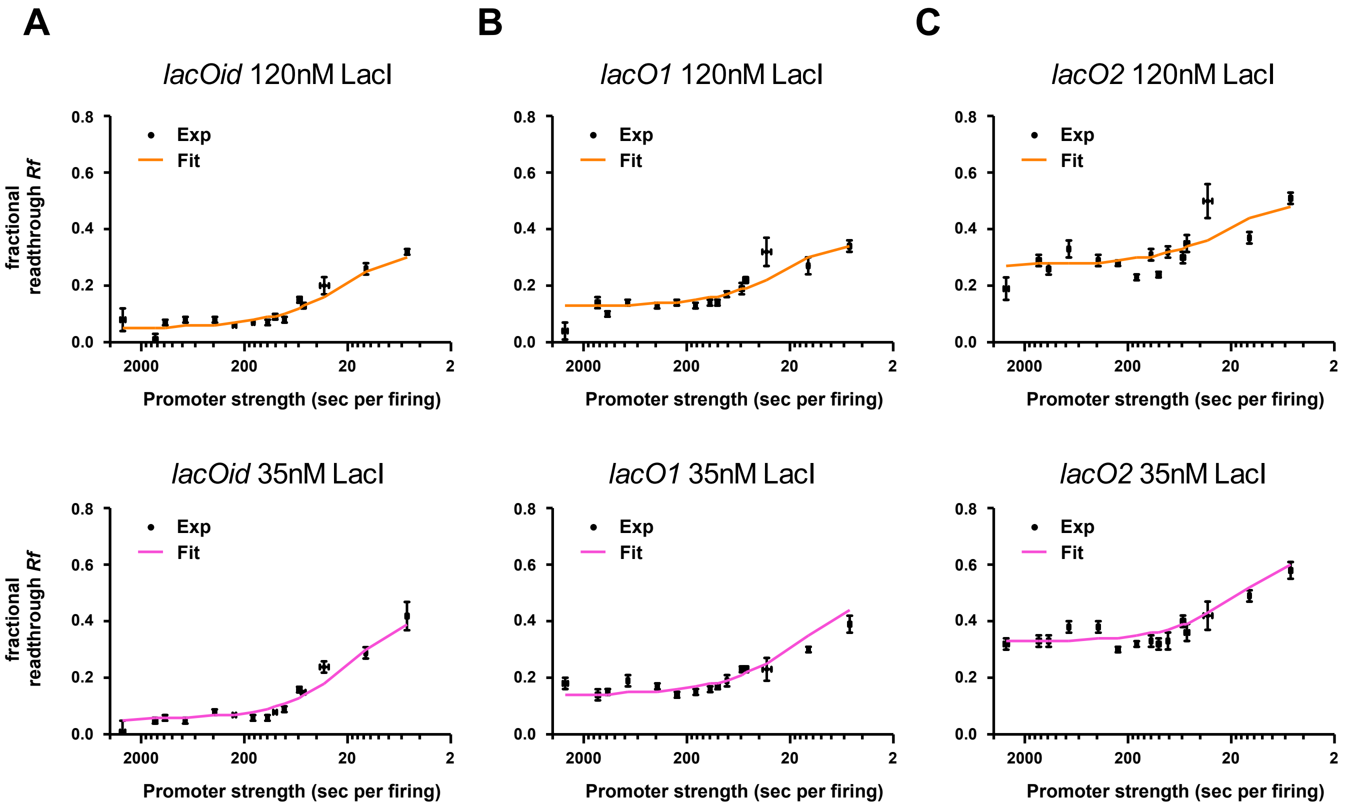

Figure S7. Global fitting. Global fitting of k_T, k_{SD}, and k_{MD} was performed for 5 LacI concentrations in combination with 3 *lac* operators. Comparisons between experimentally determined and simulated *Rf* for 3 LacI concentrations (i.e. 250nM, 70nM, and 15nM), with each of the lac operators, are shown in Figure 4A-C. The results for the remaining 2 LacI concentrations (i.e. 120nM and 35nM) are shown above. Error bars are 95% confidence intervals (n=9). Related to Figure 4.

Figure S8

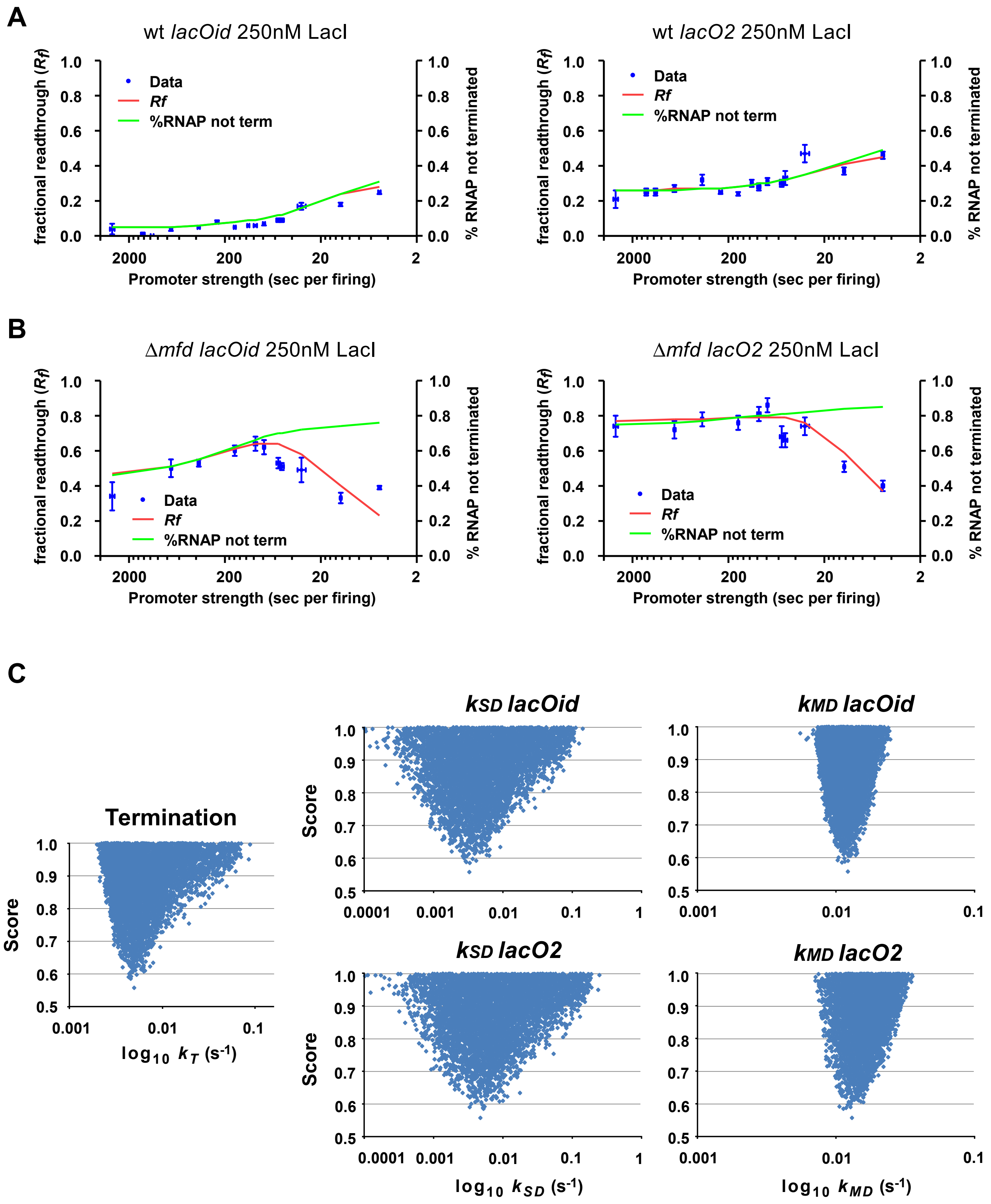

A
wt lacOid 250nM LacI
wt lacO2 250nM LacI
Data
Rf
%RNAP not term
fractional readthrough (Rf)
% RNAP not terminated
Promoter strength (sec per firing)
B
Δmfd lacOid 250nM LacI
Δmfd lacO2 250nM LacI
C
Termination
kSD lacOid
kMD lacOid
kSD lacO2
kMD lacO2
Score
log10 kT (s-1)
log10 kSD (s-1)
log10 kMD (s-1)

Figure S8. Effect of Δ*mfd* on roadblocking. A) Overlay between experimentally determined *Rf* with simulated *Rf* (red) and the fraction of RNAPs passing through the roadblock site without termination (green) in the presence of 250nM of LacI. In the roadblock model, any RNAP that has been loaded to the promoter will either pass through the roadblock to produce a transcript or be terminated. The ratio of RNAP successfully passing through a transcriptional roadblock in the presence of roadblocker protein per unit time to its absence in the same unit of time gives *Rf*. Thus, in theory, if the firing rate of the promoter is the same regardless of whether LacI is present or not, the fraction of RNAP not terminated should always match *Rf*. This is indeed what happens in the wild type cells for both *lacOid* and *lacO2*. However, in Δ*mfd* cells (B), the fraction of RNAP terminated diverges from *Rf* at high promoter firing rates. This is due to promoter clogging, in which RNAPs queue up at the roadblock site, preventing binding of further RNAP to the promoter. Thus, in this scenario, the roadblocker protein not only reduces the progression of RNAP, but also reduces the effective firing rate of the promoter. C) Global parameter fitting was performed for data obtained from Δ*mfd* cells with 2 *lac* operators (*lacOid* and *lacO2*) and 3 LacI concentrations (250nM, 70nM, and 15nM). All fitting parameters k_T, k_{SD}, and k_{MD} converge on clear optimal values. Related to Figure 5.

Figure S9

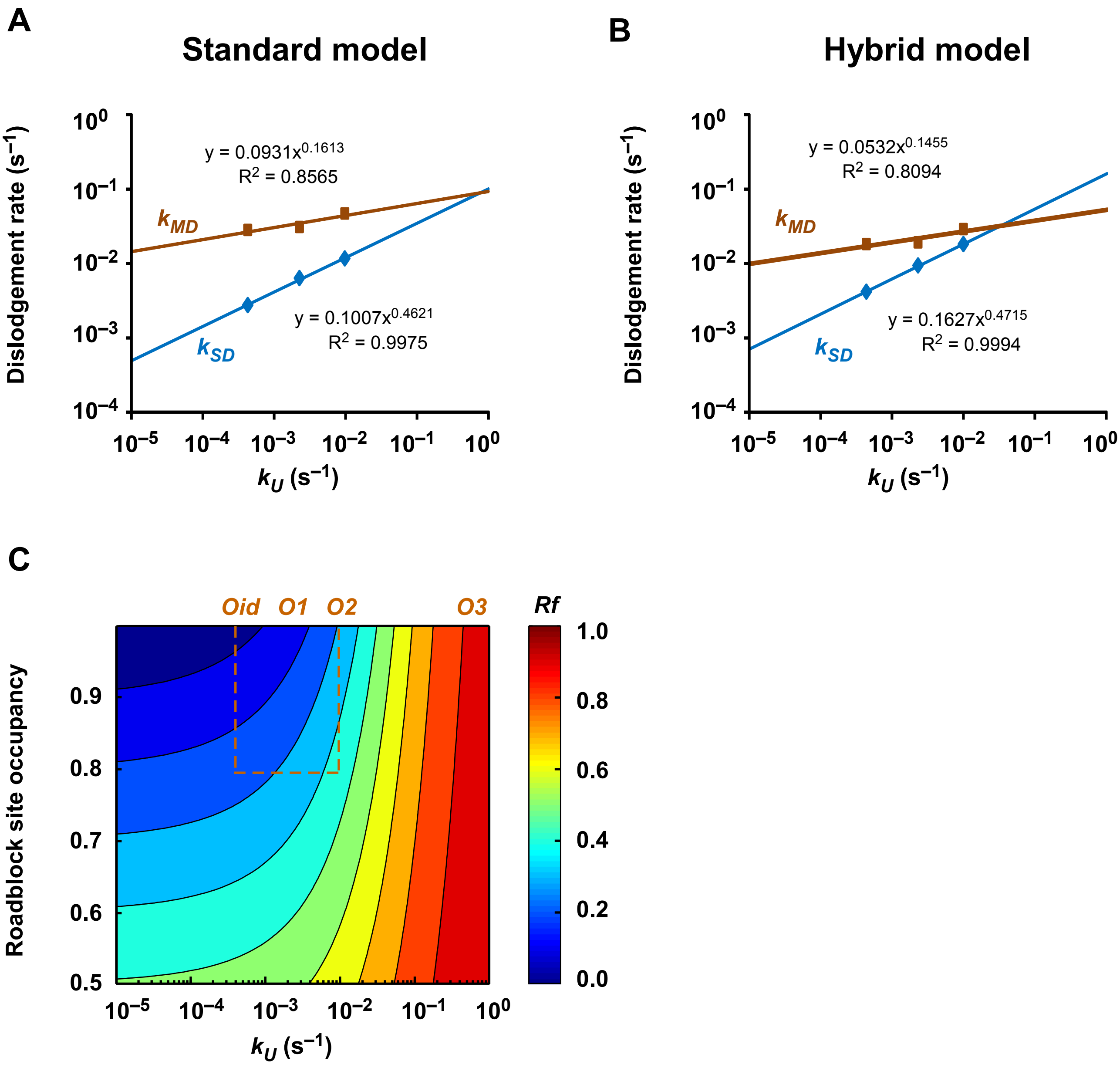

Figure S9. Extrapolating k_{SD} and k_{MD} to lower and higher operator affinities. Points show estimated values of k_{SD} and k_{MD} for *Oid*, *O1* and *O2* for the standard model (A), and hybrid termination protection model (B). Lines are power law fits. C) As shown on Figure 7, roadblocking effect of a protein binding site can be reduced while maintaining its occupancy by increasing both k_U and k_B (keeping $k_B/(k_U+k_B)$ constant). This is also true for the hybrid termination protection model (k_T=0.085). Plot was generated using the analytical equation for low RNAP flux (Figure 1D) with k_F=0 and the power law extrapolations for k_{SD} versus k_U. The boxed area shows the parameter region we examined experimentally. Related to Figure 7.
